# Determination of Eugenol Residues in Fish Tissue, Transport, and Temporary Water of Aquatic Product by Gas Chromatography–Tandem Mass Spectrometry with Application of the Electrospun Nanofibrous Membrane

**DOI:** 10.3390/foods13020238

**Published:** 2024-01-11

**Authors:** Deqian Wang, Yunning Wang, Bolin Liu, Ling Ni, Jian Zhong, Jing Xie, Zhengquan Wang

**Affiliations:** 1 College of Food Science and Technology, Shanghai Ocean University, Shanghai 201306, China; m210300950@st.shou.edu.cn (D.W.); m220300952@st.shou.edu.cn (Y.W.); bolinbyy@163.com (B.L.); lin@shou.edu.cn (L.N.); jzhong@shsmu.edu.cn (J.Z.); jxie@shou.edu.cn (J.X.); 2 Laboratory of Quality & Safety Risk Assessment for Aquatic Products on Storage and Preservation (Shanghai), Ministry of Agriculture, Shanghai 201306, China; 3 Shanghai Engineering Research Center of Aquatic-Product Processing & Preservation, Shanghai 201306, China; 4 Shanghai Key Laboratory of Pediatric Gastroenterology and Nutrition, Shanghai Institute for Pediatric Research, Xinhua Hospital, Shanghai Jiao Tong University School of Medicine, Shanghai 200092, China

**Keywords:** eugenol residues, gas chromatography–spectrometry, electrospun nanofibrous membrane, fish tissue and water analysis, food safety inspection

## Abstract

Using gas chromatography–tandem mass spectrometry and electrospun nanofibrous membrane, we developed and validated a simple, rapid, and sensitive methodology for quantifying eugenol residues in fish tissue and water samples. Fish tissue extract and water samples (315 samples) collected from three southeastern China provinces (Shanghai, Zhejiang, and Fujian), originating from eight provinces of Zhejiang, Jiangsu, Shandong, Guangdong, Fujian, Anhui, Shanghai, and Jiangxi, from April 2021 to April 2023 were filtered with an electrospun nanofiber membrane, extracted with trichloromethane/*n*-hexane, and directly concentrated to dry after simple purification. An internal standard of *p*-terphenyl in *n*-hexane and 5-µL injection volumes of the solutions was used to analyze eugenol via internal calibration with a minimum concentration of 0.5 µg/L in water samples and 0.1 µg/kg in aquatic product samples. The highest amount of eugenol was detected in Fujian province, possibly due to the higher temperature during transportation, while the lowest amount was found in Shanghai, which mainly uses temporary fish-culture devices. This is a fast, inexpensive, and effective method for testing large quantities of fish water and meat samples.

## 1. Introduction

Eugenol is a volatile phenylpropanoid formally derived from guaicol. It is a major component of clove leaves, including small amounts of isoeugenol and methyl eugenol [1]. As a naturally derived bioactive substance, eugenol exhibits anti-inflammatory, antiviral, and antimicrobial properties and is commonly used in dental clinical applications as a local antiseptic and anesthetic to relieve pain and promote healing [2,3]. Moreover, it is used as a natural pesticide, such as a fungicide, on meat and cheese to prevent salmonella colonization or as a humane method to induce deep sleep in aquarium fish and wild fish [4,5,6]. In 1972, Professor Endo, a Japanese scientist, first revealed that eugenol exhibited an anesthetic effect on various freshwater fish [7]. Today, eugenol is generally accepted as an anesthetic used before other anesthetics during transportation to slow fish metabolism. However, among the fish anesthetics, only MS-222 has been approved by the American and Canadian FDAs but is constrained by a 21-day withdrawal period. Also, MS-222 is less effective than eugenol; it is more expensive, requires a higher dose, and has a short recovery time [8,9]. Thus, clove oil (AQUI-S^®^ 20E and AQUI-S^TM^) containing active ingredients such as eugenol, isoeugenol, and methyl eugenol, has been suggested for use in aquaculture to sedate fish and reduce mortality during transportation or stocking operations. It is safer and more effective [10].

The stress level in fish markedly increases during aquaculture practices, such as handling, sorting by size, weighing, increased farming density, and transportation. These stressors can cause changes in the levels of plasma cortisol, glucose, plasma chloride, sodium, dissolved oxygen, and ammonia in transported water to induce physiological stress responses in fish [11,12,13]. Eugenol can lower ammonia and CO_2_ excretion with increasing fish mass, reduce the net Na^+^, Cl^–^, and K^+^ losses, minimize metabolic disturbance, decrease transport sensitivity, and limit the degradation of maintained water quality, thus contributing to reduced stress and mortality at high loading density during long-distance transportation [14,15,16,17]. Other advantages of eugenol include low cost, no legal regulation, easy handling, suitability for both warm and cool water fish species, and convenient removal from fish after treatment during transportation [14,15,16,18]. In developing countries like China, fish farmers, wholesalers, and retailers often use inexpensive and highly efficient anesthetics, like eugenol, for fish transportation. However, adding excessive amounts of eugenol to the transport water often results in overdosing, which requires strict and careful monitoring of the entire transportation process and end products. In contrast, developed countries emphasize fish anesthetics and request toxicological data for support. For example, anesthetic products containing eugenol (AQUI-S^®^ 20E and AQUI-S^TM^) have been legally and widely accepted by some countries, like Japan, which, in 2014, stipulated that the maximum residue limit of eugenol in fish should be 0.05 mg/kg, and the withdrawal time should be 7 days [18].

Eugenol is a noncarcinogenic natural chemical that can be easily removed and degraded in fish with fresh flowing water flush and soak [18]. However, other properties of eugenol, such as the possibility of the residue after treatment and the thermal stability of the nature or fish tissues (FT), which can adversely impact coral growth, are still a concern [14,19,20,21]. Moreover, the methylation of eugenol produces a genotoxic carcinogen, such as methyl eugenol, which is a potential hazard to human health [22]. Because of the potential risks and current status of the widespread use of eugenol anesthesia in edible fish, authorities or government regulators from most countries endorse using the residue analysis methods. Various extraction techniques for detecting eugenol, isoeugenol, and methyl eugenol in FT have been developed (Table 1); yet, all have certain limitations. For example, liquid–liquid extraction combined with nitrogen concentration is used to extract carp muscle tissue using acetonitrile (ACN), but it requires a large number of reagents [23]. The solid-phase extraction (SPE) columns are expensive for the classic residue analysis method [24]. The Pall syringe filter-assisted filtration method is too complicated and expensive for many samples [18,19]. The Quick, Easy, Cheap, Effective, Rugged, and Safe (QuEChERS) method and ultrasound-assisted extraction (UAE) method were compared for the average recovery rate and intra-day precision of eight alkylbenzenes, including eugenol and methyl eugenol. It was found that the UAE had a higher overall recovery rate and was simpler than the QuEChERS method in vegetable substrates [25]. Table 1 shows that both gas or high-performance liquid chromatography–tandem mass spectrometry (GC-MS/MS or HPLC-MS/MS) can achieve good performance for the three compounds, and the internal standard (ISTD) significantly improves the limit of detection (LOD) and eliminates the systematic errors. Considering the low cost, ease of operation, and environmental friendliness, we used GC-MS/MS plus ISTD in this study to challenge the inspection of eugenol residues in FT and corresponding water samples [18,19,20,23,24,25,26,27,28,29,30,31,32,33,34].

One crucial aspect of the pre-treatment process involves using filter membrane filtration before sample analysis, which is highly effective in reducing the LOD of the instrument and extending its operational lifespan. The existing literature has demonstrated that employing a dual-filter membrane setup in series yields superior purification results [35]. Nevertheless, when confronted with complex matrices and high-throughput sample pre-treatment, the current methods exhibited limitations, necessitating further enhancements in pre-treatment techniques for massive sample inspection, such as the commonly used 0.22 μm polytetrafluoroethylene membrane, which is easily contaminated, resulting in slow filtration speed. In recent years, more research has focused on utilizing electrospun nanofiber membranes (ENM) to treat water samples [36,37]. ENMs are particularly well-suited for the rapid filtration of liquid samples due to their substantial specific surface area, high porosity, excellent permeability, adjustable nanopore uniformity, and minimal impact on water flux. The selection of the polymer solution used in the electrospinning process has a pivotal role in shaping the morphology and properties of the resulting nanofibers [37]. High water flux and very fast filtration speed promote high throughput sampling when using ENMs [38]. Also, ENM is widely used for water treatment, including separating heavy metal ions, solution pre-treatment, solvent separation, and wastewater treatment [37,38,39]. Additionally, its biodegradability and antibacterial properties make it suitable for filtering complex matrices or water samples [39,40,41]. In the present study, the pre-treatment conditions and solvent extraction parameters were systematically optimized to cater to the analysis of FT, transport water samples (TRWS), and temporary water samples (TEWS) collected from Southeast China, an area of significant government interest in the past two years. The large-volume injection (LVI)-GC-MS/MS with ENM technique was applied to 315 samples of transport water, temporary water (TATW), and FT collected from several Chinese provinces. Suitable pre-treatment conditions, which were suitable for the detection of eugenol in aquatic matrices with good results, were applied.

## 2. Experimental

### 2.1. Reagents and Chemicals

Standard and ISTD solutions were purchased from AnPu Company (Shanghai, China) and stored at −20 °C. Trichloromethane (TCM) and *n*-hexane (HEX) were obtained from Fisher Scientific (Pittsburgh, PA, USA) and were of HPLC grade. Anhydrous sodium chloride (NaCl) and magnesium sulfate (anh. MgSO_4_) were analytical grades from Beijing Chemical Works (Beijing, China). Poly (D,L-lactide-co-ε-caprolactone) [P(DLLA-CL)] (70:30) viscosity factor 2.3 was purchased from Jinan Daigang and had an inherent viscosity of 2.3 dL/g. Dichloromethane (AR) was purchased from Sinopharm Chemical Reagent Company.

### 2.2. Sample Collection

The long-distance TRWS and TEWS, which were transported from aquaculture and mariculture fish farms to markets before the sale, and their corresponding aquatic product samples were collected from several major wholesale aquatic markets of Shanghai, Zhejiang, and Fujian provinces from April 2021 to April 2023. The collected water samples were 1 L, and the fish samples were 1.5 kg. During the transportation, several species of fish were placed on the same transport vehicle and temporarily kept in different temporary ponds upon arrival at the destination, so some fish samples shared the same transport water, similar to the temporary water samples. We ensured that all the collected fish samples corresponded to the transported water samples and the temporary water samples. All the collected samples were immediately stored at −20 °C in a car refrigerator to prevent degradation and loss of eugenol. All analysis procedures were completed within 72 h. Please refer to Table S1 and Figure S1 for more information on sample collection.

### 2.3. Apparatus

The used apparatus was a programmed temperature vaporization large-volume injection GC-MS/MS (PTV-LVI, Thermo Fisher Scientific, Taltham, MA, USA) equipped with an HP-5 MS column (30 m × 0.25 mm, 0.25 mm), electrostatic spinning machine (JDF04, Changsha Nayi Co., Ltd., Changsha, China), Inverted Optical Microscope (MS500W, Shanghai Mingzi Precision Instrument Co., Ltd., Shanghai, China), Scanning Electron Microscope (S3400, Hitachi, Tokyo, Japan), and high-voltage generator (Tianjin Dongwen High Voltage Power Supply Co., Ltd., Tianjin, China).

#### 2.3.1. GC Operation Conditions

The GC conditions were 80 °C for 1 min, 30 °C/min to 280 °C, holding for 1 min, 30 °C/min to 300 °C, and holding for 1 min; helium flow 1.0 mL/min; inlet temperature 50 °C, and injection volume 5 µL. The split flow rate was 50 mL/min for 0.05 min at 5 kPa, and the inlet was ballistically heated up to 250 °C at 14.5 °C/s and held for 1 min, followed by 300 °C for 10 min at 20 mL/min. The MS conditions involved using the default and the parent ion and secondary ion mass spectrometry acquisition parameters of eugenol and ISTD, as shown in Table 2a. Working solutions (5–200 µg/L) were prepared daily by diluting combined stock solutions with ethyl acetate and stored in the dark at 4 °C, while isoeugenol and Ter (*p*-terphenyl) as ISTD were spiked with 10 µg/L. Matrix matching of the calibration solution was performed by adding a certain amount of working solution to the blank matrix of the sample.

#### 2.3.2. Electrostatic Spinning Machine Operating Conditions

Under a 20 KV electrostatic field, the distance between the injection needle and the collector (stainless steel plate) was 10 cm; the nozzle speed was 500 mL/h; the temperature was maintained at 30–35 °C; the humidity was kept at 30–40 °C, and the needle was moved at a speed of 50 mm/min.

### 2.4. Preparation of ENM

A 100 wt% solution was prepared by weighing an amount of P(DLLA-CL) dissolved in dichloromethane with magnetic stirring (200 rpm) for 3 h. It was injected into a 5 mL syringe fitted with a needle (inner diameter of 0.41 mm) and placed on a syringe pump. A certain thickness of the spinning was obtained for 8 h film, and the thickness of the electrostatically spun nanofiber film was controlled by adjusting the feed rate and collection time. The film was removed from the stainless steel plate and cut into 6 × 6 cm square pieces for vacuum filtration of water samples. The morphology of the nanofibers was observed using an inverted optical microscope and a scanning electron microscope.

### 2.5. Procedure

**Figure 1:**
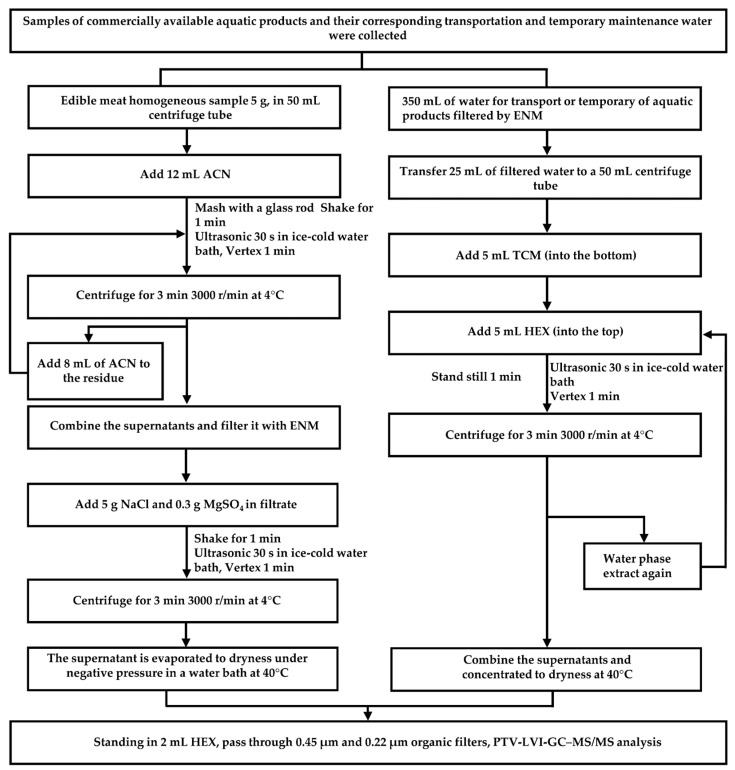
The protocol for extracting eugenol from aquatic products and their corresponding transportation and temporary maintenance water. ENM: electrospun nanofibrous membrane; TCM: trichloromethane; HEX: *n*-hexane; PTV-LVI-GC–MS/MS: programmed temperature vaporization large-volume injection gas chromatography with tandem mass spectrometry detection.

Please refer to Figure 1 for the pre-treatment steps of water and FT samples. To optimize the efficiency of this study and save time, the water samples consisted of an equal mixture of TATW (1:1 ratio). If a positive detection occurred, the corresponding samples were tested individually.

### 2.6. Method Validation

The linearity, LOD, limit of quantification (LOQ), accuracy, and precision were investigated. The linearity concentration levels ranged from 5 to 200 µg/L. The LODs (defined as the signal-to-noise ratio of 3, S/N) and LOQs (defined as the S/N of 10) for analytes were determined. The recovery and repeatability experiments were carried out on the same day. The eugenol and Ter in the samples were spiked before pre-treatment, and the recovery experiments were conducted in triplicate at an acceptable range of 70–120%. Six fortification levels of tissue were set at 5, 20, 50, 100, 150, and 200 µg/kg (*n* = 6), while six fortification levels of TATW (1:1 ratio) were set at 5, 20, 50, 100, 150, and 200 µg/L (*n* = 6). To evaluate fortified precision, the inter-day and intra-day precisions were examined. This method’s development was based on using sample blanks of the TATW (1:1 ratio) of *Channa argus*.

## 3. Results and Discussion

### 3.1. Optimization of the Extraction of Eugenol

In order to avoid matrix effects (ME) as much as possible, improvements were made to the preprocessing process. Our previous studies found that a mixed extraction solvent could improve the extraction efficiency to meet the needs of complex liquid matrices [42]. The use of TCM and HEX showed good reproducibility and NaCl and MgSO_4_ (to remove remaining water and exhibit salting-out of organic compounds) could help extract eugenol from the emulsified extract mixture [29]. By serving as a simple and instrument-free solid–liquid extraction method, UAE demonstrates advantages over the QuEChERS method in terms of simplified operational steps and higher recovery rates [25]. Additionally, UAE presents a more cost-effective alternative to traditional SPE techniques [24]. In this study, we employed Ultrasonic ice-cold water bath-assisted extraction (UIAE) [25]. UIAE could prevent the evaporation of volatiles or organic compounds from the water sample, as described in Figure 1. Moreover, a previous study suggested that a combination of ACN with NaCl and MgSO_4_ formed a biphasic system and a dry organic layer for muscle tissue extraction [43]. In this study, we added 12 mL of ACN to ensure that we would end up with 10 mL of the active ingredient, and a further 8 mL of ACN was added to the residue to save reagents (maximum extraction). In addition, for the extraction of water samples, 5 mL of TCM, HEX, and 25 mL of water samples were added to avoid splashing in the 50 mL centrifuge tubes. As a result, we found that the second filtration left almost no residue of eugenol. The samples underwent filtration through 0.45 µm and 0.22 µm organic filters for maximum purification, preceding the ‘standing in 2 mL HEX’ process, thereby extending the life of the equipment.

Given the sensitivity of the eugenol compound in the context of GC-MS, the utilization of a triple quadrupole instrument proves advantageous in mitigating interference stemming from intricate matrices compared with the LOD and LOQ of commonly used or commercially available methods (see Table 1). In this paper, we have devised a robust analytical approach for quantifying eugenol in aquatic products by capitalizing on the convergence of detection methodologies across diverse matrices. Compared with commonly used methods (see Table 1), this study dispenses with the requirement for solid-phase extraction columns, streamlining the process and conserving reagents, which offer a rapid filtration rate, high recovery efficiency, and cost-effectiveness. This method leverages an ISTD approach, incorporating an aquatic product matrix augmented by agitation and UIAE techniques. A special focus was placed on refining the pre-treatment process to ensure optimal concentration and purity of the analyte. This methodology holds considerable promise in enabling precise and reliable eugenol detection, especially within Southeast China’s sample landscape, which has recently drawn considerable attention from governmental entities.

### 3.2. Preparation and Characterization of ENM

**Figure 2:**
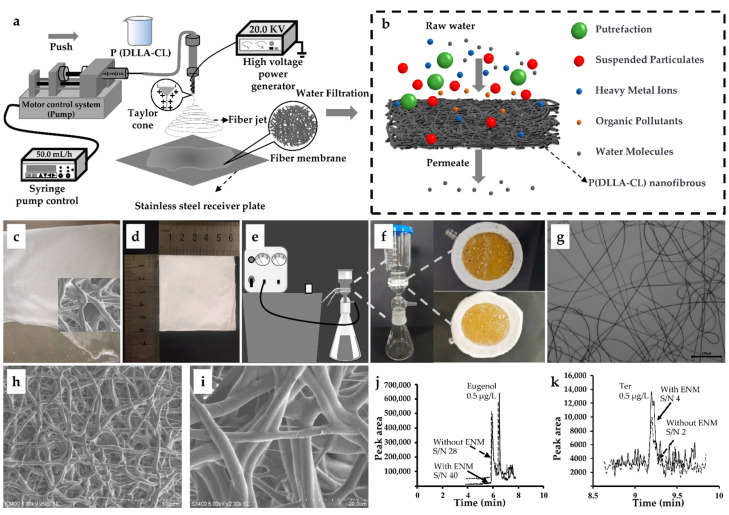
Preparation and characterization of electrospun nanofibrous membranes. (**a**) Preparation process of P (DLLA-CL) nanofibrous membranes. (**b**) Water sample filtration diagram. (**c**) Preparation of P (DLLA-CL) nanofibrous membranes on stainless steel plates. (**d**) A 6 cm polymer film. (**e**) Vacuum filtration units. (**f**) Polymer membrane after sample filtration, top view (Top: FT; Bottom: TATW). (**g**) Optical micrograph at 10% polymer concentration; (**h**,**i**) Scanning electron micrographs of Poly (D, L-lactide-co-ε-caprolactone) [P(DLLA-CL)] nanofilms. (**j**,**k**) Difference between GC-MS/MS selected reaction monitoring (SRM) mode with ENM and without ENM. FT: Fish tissue; TATW: Equal parts of transport water and temporary water combined in a 1:1 ratio; Ter: *p*-terphenyl.

Since ENMs are often thin layers, they are easily damaged when used for water filtration. Also, they cannot be reused when the mechanical strength of the material is insufficient [37]. P(DLLA-CL) has been successfully used to prepare electrostatically spun nanofiber membranes with excellent mechanical properties [44,45]. The solubility characteristics of the polymer have a pivotal role in the fabrication of nanofiber membranes [37]. A large number of particles and discontinuous fibers were produced by a 5% wt solution of P(DLLA-CL). However, a 15% wt solution of P(DLLA-CL) caused blockage of the electrospinning device’s syringe due to its high concentration, which prevented fiber production. Only a 10% wt solution of P(DLLA-CL) produced a large quantity of uniformly distributed fibers (Figure 2). The interplay of temperature and humidity is a pivotal factor for the successful electrospinning of nanofiber membranes, with this investigation drawing insights from earlier works [35,37]. Additionally, the distance between the spinneret tip and the collector significantly influences nanofiber morphology [38]. Previous studies suggested that a distance of 15 cm is more suitable for this process as it resulted in a uniform nanofiber membrane with minimal particle presence. The remaining parameters of the electrospinning apparatus adhered to prior protocols [35]. Although preparing ENMs requires significant time, the process is simple and can be adapted to large-scale production. The final product can successfully filter impurities such as deteriorated substances, particulate matter, macromolecular viruses, organic compounds, and heavy metals from samples [39].

This study used an electrostatic spinning technique to prepare electrostatically spun nanofibrous P(DLLA-CL) membranes with dichloromethane as solvent (Figure 2a,b). Observation by optical microscope revealed nanofibers (Figure 2g). The membranes were cut into approximately 6 cm squares (Figure 2c,d) and then used for filtration in the pre-treatment process (Figure 1 and Figure 2b–f). A large number of P(DLLA-CL) fibers were successfully prepared, as observed by scanning electron microscopy (Figure 2h,i). Conventional filtration methods for fish tissue samples are easily clogged and difficult to pre-treat. Especially for large amounts of sample processing, it is usually necessary to change the filtration membrane, which is a slow, cumbersome, and error-prone process. In this study, we prepared an ENM for the filtration of fish tissue homogenates, which does not clog during filtration of fish tissue homogenates. Through experiments, it was found that the filtration rate of ENM was dozens of times higher than that of traditional membranes, and the increase in impurities filtered on the membrane was visible to the naked eye, mainly due to the high porosity and physical adsorption function of ENM. Due to the very high mechanical strength of P(DLLA-CL) and its reusability [36], no significant decrease in filtration rate was detected, and the filtrate was clarified. ENM prepared by P(DLLA-CL) can effectively filter the impurities in water samples with high speed and excellent mechanical properties; it is not easy to deform and rupture, it is biodegradable and non-polluting, reusable, and easy to recycle and replace. The characterization of ENM is shown in Figure 2. ENM was used and compared, which showed better performance on S/N and, thus, was used (Figure 2j,k). Both the peaks of eugenol and Ter were diminished; however, the recovery ratio increased by 30% after using ENM, representing a high efficiency for purification and assay.

### 3.3. Optimization of ISTD

Using homologs to the target compound as ISTDs is a common method to eliminate ME [46]. Although accurate analytical methods must be utilized in the certified reference materials as standard (e.g., using isotopic ISTDs has been recognized as a primary measurement method), the high price of isotopic ISTDs is unsuitable for economically underdeveloped regions and institutions. Isomers or other substitutes of standard materials can effectively reduce the cost of large-scale food safety screening by government agencies and meet their basic needs for rapid and large-scale testing [46]. At the beginning of this study, an ISTD method for the detection of eugenol residues in aquatic products by triple quadrupole gas chromatography was established, and the isoeugenol was selected as the ISTD in case of the complexity of the matrix of aquatic products and environmental water samples. However, subsequent long-term experiments revealed that the isoeugenol standard is very unstable and may be easily decomposed. Although the recovery and precision of isoeugenol as an ISTD method were satisfactory, the instability of the ISTD greatly impacted the quantification of eugenol. Deuterated eugenol, such as eugenol-d_3_ or ^14^C-eugenol, could better simulate the metabolic pathway of eugenol in the matrix than Ter but is more expensive and less stable [18,23]. Therefore, we have chosen the stable and inexpensive ISTD Ter, a commonly used ISTD for volatile phenolic compounds residue analysis, as the ISTD for subsequent research.

**Figure 3:**
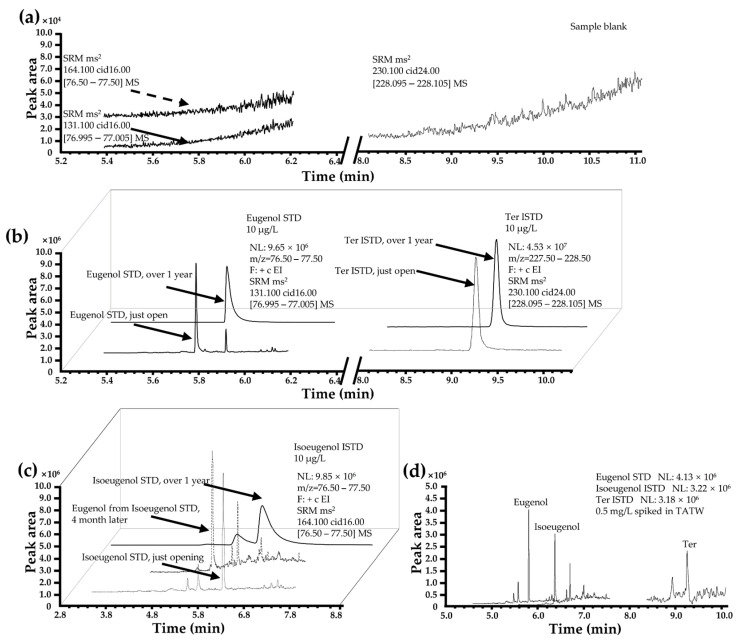
Extracted ion chromatogram (EIC) of (1) eugenol, (2) isoeugenol, and (3) *p*-terphenyl analysis by SRM mode. (**a**) EIC of (1), (2), and (3) from sample blank. (**b**) Eugenol standard solution is not converted into isoeugenol from 4 months to 1 year. (**c**) Isoeugenol standard solution is partly converted into eugenol from 4 months to 1 year. (**d**) A spike experiment (1), (2), and (3) in the TATW of *Channa argus*.

After storage at −20 °C for 4 months, the ISTD of Ter was superior to that of isoeugenol (Figure 3b–d). Under such conditions, the standard solution of eugenol did not contain isoeugenol for 1 year, but some isoeugenol converted into eugenol in the standard solution of isoeugenol stored for 4 months and 1 year (Figure 3c). ISTD of Ter was chemically stable for 1 year in HEX and could be easily applied in TATW (1:1 ratio) and FT matrix (Figure 3a–d).

### 3.4. Optimization of Injection Mode

**Figure 4:**
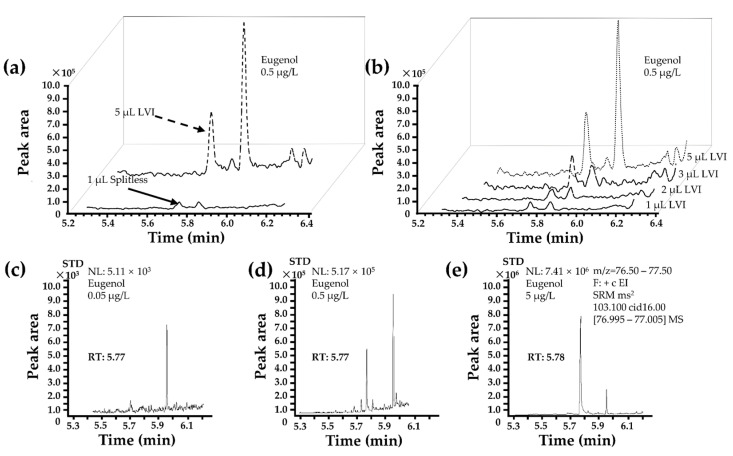
(**a**) Compare the injection mode of LVI and splitless. (**b**) Optimization of LVI injected ion volume. (**c**) EIC of eugenol from the mixture TATW of six different matrix blanks of aquatic product samples. (**d**) EIC of eugenol spiked at 0.5 µg/L in the mixture TATW of six kinds of aquatic product samples under optimization by SRM mode. (**e**) EIC of eugenol spiked at 5 µg/L in the mixture TATW of six aquatic product samples under optimization by SRM mode.

By adjusting the injection method after identifying Ter as an ISTD, the sensitivity of this method was improved, making it more suitable for the analysis of a large number of real samples and increasing the detection sensitivity without increasing the number of pre-treatment steps and time, thus allowing for it to be combined with LVI. The preprocessing method remained largely unchanged after optimization. Before sample loading, the final volume was adjusted to 2 µL to accommodate the dual-filter membrane tandem purification. The sample injection volume was progressively increased from 0.5, 1, 2, and 3 to 5 µL. Eventually, it was confirmed that 5 µL served as the optimal injection volume. While the chromatographic and mass spectrometric conditions remained mostly constant, the injection volume was expanded to 5 µL (Figure 4a,b).

Ter coupled with LVI induced good linearity and repeatability, which was eight times better than the splitless injection mode (Figure 4a,b and Table 2b). Therefore, Ter was further used to optimize this method and the recoveries of six different concentrations for ISTD (Table 2c, 10 µg/L). The results showed that the relative standard deviation (RSD, %) was <8%. In addition, 5 µL injection volumes (depending on Thermo LVI-GC-MS/MS system, 10 µL maximum syringe range) for the PTV-LVI technique induced a dose-dependent improvement in eugenol responses and sharply decreased the signal-to-noise ratio (S/N) to 2.3. Hence, this injection volume was selected and optimized. For the LVI method, the linear regression equation was Y (peak area) = −0.0617 + 0.1208X (concentration) with an r^2^ of 0.9988, LOD of 0.5 µg/L, LOQ of 1.5 µg/L, and a (S/N) of 2.3 (Table 2). The LOD for the fish sample was 0.1 µg/kg, and the LOQ was 0.5 µg/kg. The S/N of the LVI method was significantly better than that of the conventional method, and the linearity was also better.

In order to verify the stability and reliability of the large volume injection method, 13 sets of blank spiking experiments were designed, and the results showed that the recoveries of 13 consecutive spiked samples were >93%, with an average recovery of 100% and an RSD of 3.68%.

### 3.5. Matrix Effect

Extraction efficiency was enhanced by optimizing the extraction procedure using a mixed extraction solvent, resulting in improved recovery of eugenol and combating ME. UIAE was employed to prevent the evaporation of volatile substances or organic compounds in the water samples, preventing potential ME. Applying an ENM membrane eliminated interfering impurities, effectively mitigating ME. A cost-effective and efficient ISTD, Ter, was employed for calibration to minimize the impact of ME. Various concentrations of matrix samples were tested, and their recovery rates were compared to those obtained from matrix samples spiked with eugenol, providing a comprehensive assessment of ME.

To verify the quantitative determination method supporting exposure studies, an investigation of ME is necessary due to the complex matrix background of fish samples, which makes accurate measurement of drug residues difficult [26]. The evaluation of ME involves comparing the chromatographic peak areas of spiked samples after extraction with those of standard solutions prepared at the same concentration in pure solvent. The calculation formula for ME is (peak area of standard substance in the matrix—peak area of standard substance in the solvent)/peak area of standard substance in the solvent ∗100%, where an ME value within ±20% indicates no significant matrix effect [23,27]. Both FT and TATW samples need to be evaluated for ME. In this study, three analyte levels (at concentrations of 10, 20, and 50 µg/g for each analyte) were added to the samples, and three replicates were performed using the proposed method. The recovery of analytes in samples was compared with that in matrix samples at the same concentration levels, and the results showed a slight enhancement in the signal of eugenol but no impact on the quantitative results of eugenol, as shown in Table 3.

The EICs of mixture TATW of six different matrix blanks of fish samples, 0.5 µg/L spiked eugenol representing LOD, and 5.0 µg/L spiked eugenol representing linear minimum concentration, are shown in Figure 4c–e. We studied six different types of aquatic products, including freshwater fish, marine fish, freshwater shrimp, marine shrimp, marine shell, and freshwater shell Chinese hairy crab. The studied species included *Channa argus*, *Pelteobagrus fulvidraco*, *Penaeus vannamei*, *Macrobrachium rosenbergii*, *Scallop*, and *Procambarus clarkia*. We tested and mixed the TATW and FT of these six different substrates. The precision of this method (repeatability and reproducibility) was evaluated using RSDs with FT samples and TATW samples fortified with eugenol. Intra-day variability (repeatability) was assessed by analyzing six spiked samples measured six times within a single day. Inter-day variability (reproducibility) was achieved by analyzing six replicate samples over six consecutive days. The RSD (%) values for inter-day and intra-day precisions were obtained in the range of 0.4–9.9% and 0.3–14.9% (10, 20, and 50 µg/L), as shown in Table 2d. Moreover, the recovery ratio (%) and variability (RSD, %) of the selected method showed a better performance in a linear range of 5–200 µg/L of the spiked sample, ranging from 97.6–104.9% and 2.1–7.2%, respectively, compared with the previous recovery rates of 76.45–109.8% (Table 1). The results described in this study are superior to the GC/MS method described in reference [31].

### 3.6. Inspection of Eugenol in Real Samples

Market research has found that if eugenol was added to aquatic products during transportation, residues of eugenol would be present not only in the FT but also in the TATW. In some cases, eugenol was not detected in FT, but it was detected in the TATW, which suggested that eugenol was used in the aquatic product. The developed method was applied to determine eugenol residue in 315 FT samples and their corresponding TATW samples collected from ten aquatic retail markets or aquaculture fields in three provinces in China over the past two years. The results showed 36 positive FT samples, 26 positive transportation water samples, and 9 positive transient water samples (Table 4a). Interestingly, we observed positive detections of eugenol in FT samples, TRWS, as well as TEWS within the same batch. This indicates that eugenol was used throughout the stages of aquaculture, transportation, and temporary holding of the fish batch. Furthermore, among the remaining positive sample batches, we found that eugenol usage occurred in one or two stages out of aquaculture, transportation, and temporary holding. Table 4a–c presents affirmative findings from eight provinces: Zhejiang; Jiangsu; Shandong; Guangdong; Fujian; Anhui; Shanghai; and Jiangxi. The table furnishes comprehensive insights into the number and proportion of positive samples within each batch from every province. Additionally, it offers details encompassing the standard error of mean differences and ranges of positive concentrations, accompanied by the distribution of samples across diverse concentration levels. Of note, Jiangxi Province exhibited the highest incidence of positive fish samples, registering a detection rate of 33.3%. In contrast, Shandong and Shanghai recorded identical detection rates, sharing the lowest rate of 3.3%. Turning to positive TRWS, Fujian Province emerged with the highest detection rate at 19.4%, whereas Shanghai reported the lowest rate (4.7%). Regarding transient water samples, Shandong Province showcased the highest positivity rate at 11.1%, whereas Fujian Province displayed the lowest rate of 2.8%.

Among the six types of aquatic products that were initially selected, positive results for eugenol were detected in *Channa argus*, *Pelteobagrus fulvidraco*, and *Penaeus vannamei*. We identified a direct correspondence between FT, transport water, and temporary water. In our sampling conducted in Zhejiang province in June, we detected positive results for eugenol in both FT samples, TRWS, and TEWS. This finding indicates the use of eugenol during the stages of aquaculture, transportation, and temporary holding. Additionally, we found variabilities in detecting eugenol in the same fish species between different batches. For example, in the Zhejiang market, positive detection of eugenol was found in the FT of *Channa argus* in June, while in August, positive detection was detected in the transport water. This indicates that eugenol was used in the breeding stage for *Channa argus* in June and during transportation in August. Additionally, positive detection of eugenol was found in the temporary water for *Channa argus* in the Shanghai market. In the Zhejiang market, positive detection of eugenol was only found in the temporary water for *Pelteobagrus fulvidraco*, while in the Shanghai market, there was positive detection in the transportation water. This suggests that eugenol was used in the temporary stage in the Zhejiang market and during transportation in the Shanghai market. As for *Penaeus vannamei*, positive detection was only found in the transportation water. In the markets of Shanghai, Zhejiang, and Fujian, it has been observed that Fujian province utilized the highest amount of eugenol, whereas Shanghai utilized the least. This discrepancy may be attributed to the highest temperatures in Fujian province, leading to higher demand for eugenol, whereas the Shanghai market relies predominantly on external supply sources, resulting in relatively lower local demand for eugenol. A direct correspondence between FT, transport water, and temporary water is rarely found, which may be due to replacing transport water with new water in the latter half of the transportation process.

**Figure 5:**
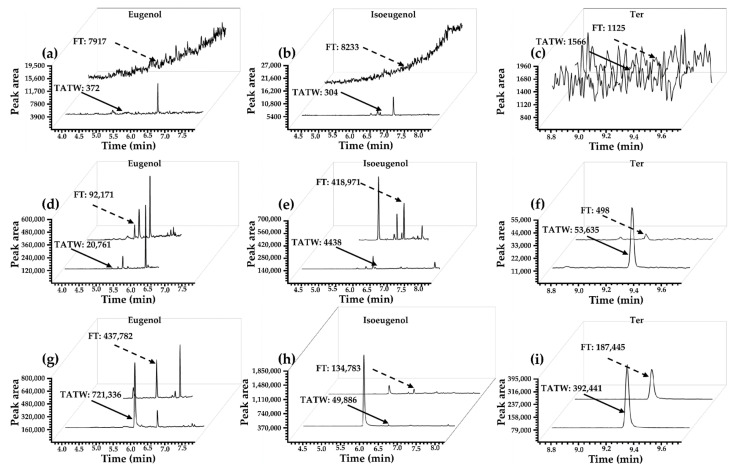
(**a**–**c**) Sample blank of FT and TATW by LVI mode. (**d**–**f**) FT (0.1 µg/kg) and TATW (0.5 µg/L) by splitless injection mode. (**g**–**i**) The 0.5 µL matrix samples of FT (0.1 µg/kg) and TATW (0.5 µg/L) by LVI mode.

Details of inspection results of origin and location are also shown in Table 4b,c, and the distributions of positive samples were also described. The results indicated that eugenol might be a risk transferred through food chains in East and South China, and this method was proven to be a simple method for screening eugenol residue in transport water, environment water samples, and FT by applying the ENM through the LVI-GC-MS/MS method. Comparison data of eugenol, isoeugenol, and Ter detection in FT and TATW based on the ENM optimized LVI-GC-MS/MS method with the traditional method are shown in Figure 5. For more results on the concentration of positive samples in different months and the frequency of positivity in different fish species, please refer to Figures S2 and S3.

## 4. Conclusions

Based on the polymeric material P (DLLA-CL), we prepared an ENM to filter an FT homogenate, TRWS, and TEWS that did not clog during sample filtration. This film has fast filtration speed and high mechanical strength, making it suitable for repeatedly filtering large samples. It exhibits better performance in terms of S/N and improves the efficiency of eugenol purification and determination. By employing a combination of mixed solvent extraction and UIAE, we successfully improved the extraction efficiency of eugenol. In our study, we identified a cost-effective ISTD, Ter, and determined that an injection volume of 5 μL was suitable for large-scale quantitative analysis of eugenol. The final results demonstrated a significant reduction in the detection limit. A simple, rapid, and sensitive method for detecting eugenol residues in water for long-distance transportation of aquatic products, temporary water, and FT has been developed by combining LVI-GC-MS/MS with ENM.

We observed positive detections of eugenol in FT samples, TRWS, as well as TEWS within the same batch, which indicates a direct correspondence between FT, transport water, and temporary water. By examining samples from different provinces in China, the highest use of eugenol was found in Fujian Province, while the lowest was in Shanghai. This may be because Fujian Province has the highest temperature among all the provinces surveyed and, therefore, has a higher demand for eugenol during transportation. On the other hand, the Shanghai market relies mainly on external sources of supply and has a relatively low demand for eugenol. In addition, this method is not affected by matrix interference; it has high sensitivity and good precision, with intra-day and inter-day variabilities of 0.4–9.9% and 0.3–14.9%, respectively. This method demonstrates good repeatability, linearity, and recovery rates in the identification, quantification, and residue analysis of eugenol, with low detection and quantification limits, thus promoting the government’s regulation of eugenol.

## Figures and Tables

**Table 1:** Summary of the studies related to eugenol, isoeugenol, and methyl eugenol determination.

Sample Amount	Compounds	Internal Standard ^a^	Technique ^b^	Sorbent ^c^	Solvent	Equipment	LODs	LOQs	Recovery (%)	Comments	Ref.
Clove oil/1 g	Eugenol	*a* -	LLE	-	MT	HPLC-UV	25.0 μg/L	50.0 μg/L	>85.0	From formulation	[20]
Fish Shrimp tissue/2 g	Eugenol	Eugenol-d_3_	SIDA+SPE +UE	-	ACN, EA	GC-MS/MS	2.5 μg/kg	5.0 μg/kg	94.7–109.8	Method development	[23]
Fish/2 g	Eugenol	-	SPE	-	MT/H_2_O (1:9, *v*/*v*)	GC-MS/MS	2.5 μg/kg	5.0 μg/kg	94.8–103.6	Method optimization for fish	[26]
Fish fillet/2 g	Eugenol +isoeugenol methyl-eugenol	-	SPE	-	EA; HEX	GC-MS	0.4, 1.2, 0.2 μg/kg	1.2, 4.0, 0.7 μg/kg	76.4–99.9	Eugenol in fish	[27]
Fish/5 g	Eugenol+ isoeugenol	-	LLE+SPE	Dry ice	AC	HPLC-UV	4.0 μg/kg	12.0 μg/kg	80.0–105.0	Stability of eugenol	[24]
Rainbow trout fillet/0.5 g	Eugenol	^14^C-eugenol	SPE+UE	Dry ice	AC, MT/H_2_O (9:1, *v*/*v*)	GC-MS	-	20.0 mg/kg	87.3–95.1	Not for residue analysis	[18]
Smoked sausages, Smoked fish/0.5 g	Eugenol +isoeugenol,	-	Mb ME	chloride acts	HEX	HPLC-UV	0.6 μg/kg	2.0 μg/kg	70.0–80.0	Smoked food	[32]
Water/10 mL; Fish fillet/5 g	Isoeugenol	-	SPE	-	MT/H_2_O (9:1, *v*/*v*)	HPLC-UV	30.0 μg/kg	90.0 μg/kg	80.0–105.0	Water and fish both	[19]
Water/5 mL	Eugenol	-	SP; SPE	-	AC	GC-MS	2.3 μg/L	7.7 μg/L	87.0–104.0	Compare SP and SPE	[28]
Rat plasma/200 μL	*cis*-methyl isoeugenol	1-naphthol	SPME; LLE	-	MT; AC	HSSPME-GC/MS	-	7.6 μg/L	80.1–89.0	Application in plasma	[29]
Serum/200 μL	Eugenol	DCHM	SPME	-	ET	HPLC-MS/MS	3.2 μg/L	4.8 μg/L	-	HS used	[30]
Rat plasma/50 μL	Eugenol	Thymol	LLE	Formic acid	AC	GC-FID/MS	-	100.0 μg/L	-	Method for plasma	[31]
NP and ME from EOAC/1 mL	Methyl-eugenol	-	HS-SPME	NaCl +Na_2_SO_4_	DCM, HEX, MeOH	GC-FID/MS	0.16 μg/mL	0.4 mg/L	>90.0	On skin samples	[33]
Eugenol Standard/0.1 mM	Eugenol	-	APTMS- GO@SnO_2_/CPE	-	-	FTIR, SEM-EDX, XRD, BET	-	-	>95.0	Standard eugenol	[34]
Pepper samples/1 g	Eugenol, methyl-eugenol	DCHM	LLE+UE	Citrate salts +NaCl+MgSO_4_	AcOEt, ACN	GC-HRMS-Q-Orbitrap	0.01 mg/kg	0.2 mg/kg	88.0–102.0	Alkenylbenzenes in pepper and its varieties	[25]
Water/25 g	Eugenol	Ter	LLE+UE	NaCl+MgSO_4_	TCM, HEX	LVI-GC-MS/MS	0.5 μg/L	1.5 μg/L	97.6–104.9	This study	
Fish fillet/5 g	Eugenol	Ter	LLE+UE	NaCl+MgSO_4_	ACN	LVI-GC-MS/MS	0.1 μg/kg	0.5 μg/kg	92.3–111.2	This study	

^a^ No record; ^b^ SPE, solid-phase extraction; SPME, solid-phase microextraction; SIDA, stable isotope dilution assay; LLE, liquid-to-liquid extraction; FID, flame ionization detector; SP, spectrophotometer; SE, Soxhlet extraction; UE, ultrasonic extraction; HSSPME-GC-MS, rapid headspace solid-phase microextraction–gas chromatography–mass spectrometry; LVI, large-volume injection; MbME, membrane-based microextraction; HS-SPME, headspace–solid-phase microextraction; GC-FID/MS, gas chromatograph with flame ionization detector; APTMS-GO@SnO_2_/CPE, aminopropyltrimethoxysilane–graphene oxide/the carbon paste electrode; FTIR, fourier transform infrared spectroscopy; SEM-EDX, scanning electron microscope–energy dispersive X-ray spectroscopy; XRD, phenompro-x, and X-ray different analysis; BET, brunauer emmert teller; GC-HRMS-Q-Orbitrap, gas chromatography–high resolution mass spectrometry–quadrupole-Orbitrap; ^c^ AC, acetone; TCM, trichloromethane; HEX, *n*-hexane; ACN, acetonitrile; MT, methanol; EA, ethyl acetate; ET, ethanol; Ter, *p*-terphenyl; DCM, dichloromethane; AcOEt, Ethyl acetate; DCHM, dicyclohexylmethanol; NP, 1-nitro-2-phenylethane; ME, methyleugenol; LOD, limit of detection; LOQ, limit of quantification.

**Table 2:** (**a**) GC-MS/MS acquisition parameters with ion transitions, collision energy, and retention time. (**b**) Comparison of splitless and LVI modes using GC-MS/MS with ENM. (**c**) RSD (%) of eugenol on six different concentrations for LVI modes using an ISTD method with ENM. (**d**) Inter-day and intraday precisions of eugenol on different concentrations for LVI modes using the TATW and FT of six kinds of aquatic products with ENM.

**(a)**						
**Compound**	**t_R_** **(min)**	**Precursor Ion (*m*/*z*)**		**Product Ion (*m*/*z*)**	**Collision Energy (eV)**	**Dwell Time (s)**
Eugenol	5.8	164.1		77.1	26	4.0
		131.1		77.0	24	4.0
		103.1		77.0	16	4.0
Isoeugenol	6.4	164.1		149.1	12	4.0
		149.1		77.0	20	4.0
		103.1		77.0	12	4.0
*p*-Terphenyl	9.3	230.1		228.1	24	1.0
		229.1		228.1	10	1.0
		228.1		226.1	26	1.0
**(b)**						
**Name**	**Splitless** **Ter as ISTD *^a^***				**LVI** **Ter as ISTD**	
**Equation**	**Y (peak area) = −0.2605 ** **+ 0.1769X (concentration)**				**Y = −0.0617** **+ 0.1208X**	
r^2^ *^b^*	0.9918				0.9988	
LOD *^c^*	4 µg/L				0.5 µg/L	
LOQ *^d^*	13 µg/L				1.5 µg/L	
S/N *^e^*	26.9				2.3	
Response ^*f*^	Low				High	
**(c)**						
**Spiking Level (µg/L) *^g^***			**Ter as ISTD**			
			**Value ± SD *^h^* (µg/L)**			
5.0			5.2 ± 0.3			
20.0			20.0 ± 0.9			
50.0			48.8 ± 1.1			
100.0			104.9 ± 3.8			
150.0			151.8 ± 8.0			
200.0			198.0 ± 14.4			
**(d)**						
**Matrix** **TATW/FT**	**RSD (%) ** **(Intra-Day Variation, *n* = 6)**			**RSD (%) ** **(Inter-Day Variation, *n* = 6)**		
	**10 *^i^***	**20**	**50**	**10**	**20**	**50**
*Channa argus*	3.6/3.4	6.7/2.8	1.5/1.6	3.1/6.2	7.6/5.5	0.4/3.2
*Pelteobagrus fulvidraco*	10.0/2.8	7.6/3.1	5.3/1.9	6.3/5.9	3.7/6.2	1.9/3.7
*Penaeus* *vannamei*	13.5/4.9	11.1/8.8	2.0/0.9	8.3/10.5	1.0/9.3	7.5/1.7
*Macrobrachium* *rosenbergii*	7.4/6.8	10.6/1.3	5.9/4.2	8.3/4.2	9.9/5.6	1.8/3.1
*Scallop*	4.5/1.5	8.6/1.9	9.0/1.1	7.7/2.4	9.2/3.5	2.0/3.0
*Procambarus* *clarkii*	8.3/3.3	14.9/5.0	0.3/0.9	5.1/4.9	5.2/8.5	8.7/0.7

ISTD *^a^*: internal standard; r^2 *b*^: correlation coefficient; LOD *^c^*: limit of detection; LOQ *^d^*: limit of quantification; S/N *^e^*: ratio at 2.0 µg/L spiked level in the transport water sample; Response *^f^*: response of eugenol at 20.0 µg/L spiked level; Spiking level (µg/L) *^g^*: spiking level of eugenol; Value ± SD *^h^* (%): value ± standard deviation; 10 *^i^*: concentration, µg/L; FT: fish tissue; TATW: equal parts of transport water and temporary water combined in a 1:1 ratio.

**Table 3:** Study of Matrix Effects (ME) in the samples spiked at different concentrations.

			Mean Recovery ± Standard Deviation (*n* = 3)				
	Spiked with Analyte (µg/L)	*Channa* *argus.*	*Pelteobagrus.* *fulvidraco*	*Penaeus.* *vannamei*	*Macrobrachium.* *rosenbergii*	*Scallop.*	*Procambarus.* *clarkii*
Matrix water	10 *^a^*	97.2 ± 4.3	92.3 ± 2.1	99.0 ± 4.3	97.5 ± 4.3	99.1 ± 3.3	97.1 ± 7.4
(µg/L)	20	96.3 ± 3.2	97.5 ± 3.3	97.5 ± 4.6	97.6 ± 3.6	100.3 ± 3.5	98.2 ± 3.9
	50	100.0 ± 4.6	97.6 ± 5.8	95.7 ± 5.2	95.3 ± 4.9	100.2 ± 2.1	99.4 ± 4.4
ME (%)		7.3	21.3	9.2	18.4	17.3	17.0
Fish tissue (µg/kg)	10	103.1 ± 6.0	98.2 ± 5.9	98.1 ± 2.6	102.1 ± 3.1	99.6 ± 5.9	97.5 ± 7.6
	20	96.4 ± 4.6	95.1 ± 3.8	98.1 ± 3.4	97.6 ± 6.3	97.7 ± 3.5	97.3 ± 2.2
	50	102.7 ± 7.1	99.0 ± 4.6	97.4 ± 5.3	97.3 ± 4.5	97.2 ± 4.2	97.8 ± 3.1
ME (%)		10.23	0.4	9.7	8.0	3.4	0.7

*^a^*: All samples were spiked with each analyte at a concentration of 10, 20, and 50 µg/L.

**Table 4:** Inspection results of eugenol residues in TRWS, TEWS, and FT from April 2021 to April 2023. (**a**) Origin distribution of positive samples. (**b**) Market distribution of positive samples. (**c**) Region distribution of positive samples.

**(a)**																
**Market**	**Type**	**Samples,** **n**	**Positive Samples,** **n (%)**	**Concentration (** **μg/L)**						**Concentration Distribution of Samples (** **μg/kg/** **μg/L), n (%)**						
				**Mean**	**±**	**SEM**	**Range**			**5–50**		**50–100**		**100–1000**		**>1000**
Zhejiang	AQS (μg/kg)	74	8(10.8)	7271.6	±	4190.1	1.1	–	32,668.8	2(2.7)		1(1.4)		2(2.7)		3(4.1)
	TRWS (μg/L)	48	40(8.3)	352.8	±	337.8	11.8	–	1366.3	3(6.3)		0(0.0)		0(0.0)		1(2.1)
	TEWS (μg/L)	32	30(9.4)	12.3	±	6.2	0.1	–	20.0	3(9.4)		0(0.0)		0(0.0)		0(0.0)
Jiangsu	AQS	49	40(8.1)	10,318.0	±	8648.1	243.0	–	36,090.3	0(0.0)		0(0.0)		2(4.1)		2(4.1)
	TRWS	40	5(12.5)	84.0	±	64.1	0.0	–	337.0	3(7.5)		1(2.5)		1(2.5)		0(0.0)
Shandong	AQS	30	10(3.3)	4678.4	±	0.0			4678.4	0(0.0)		0(0.0)		0(0.0)		1(3.3)
	TEWS	9	1(11.1)	13.2	±	0.0			13.2	1(11.1)		0(0.0)		0(0.0)		0(0.0)
Guangdong	AQS	36	4(11.1)	3107.6	±	3011.4	53.1	–	12,141.6	0(0.0)		2(5.6)		1(2.8)		1(2.8)
	TRWS	16	10(6.3)	648.0	±	0.0		–	648.0	0(0.0)		0(0.0)		1(6.3)		0(0.0)
Fujian	AQS	81	15(18.5)	4692.1	±	2196.0	0.4	–	30593.6	4(4.9)		1(1.2)		3(3.7)		7(8.6)
	TRWS	67	13(19.4)	20.8	±	6.8	0.0	–	89.2	12(17.9)		1(1.5)		0(0.0)		0(0.0)
	TEWS	36	10(2.8)	25.4	±	0.0			25.4	1(2.8)		0(0.0)		0(0.0)		0(0.0)
Anhui	AQS	9	1(11.1)	163.0	±	0.0			163.0	0(0.0)		0(0.0)		1(11.1)		0(0.0)
	TRWS	11	10(9.1)	993.0	±	0.0			993.0	0(0.0)		0(0.0)		1(9.1)		0(0.0)
Shanghai	AQS	30	10(3.3)	109.0	±	0.0			109.0	0(0.0)		0(0.0)		1(3.3)		0(0.0)
	TRWS	43	20(4.7)	177.0	±	177.0	0.0	–	354.0	1(2.3)		0(0.0)		1(2.3)		0(0.0)
	TEWS	57	40(7.0)	10.0	±	4.5	0.0	–	18.1	4(7.0)		0(0.0)		0(0.0)		0(0.0)
Jiangxi	AQS	6	2(33.3)	145.9	±	78.2	67.7	–	224.0	0(0.0)		1(16.7)		1(16.7)		0(0.0)
	TRWS	7	1(14.3)	19.6	±	0.0		–	19.6	1(14.3)		0(0.0)		0(0.0)		0(0.0)
Total	AQS	315	36(11.4)	5208.3	±	1609.8	0.4	–	36,090.3	6(1.9)		5(1.6)		11(3.5)		14(4.4)
	TRWS	247	27(10.9)	152.5	±	64.3	152.5	–	64.3	20(8.1)		2(0.8)		4(1.6)		1(0.4)
	TEWS	165	90(5.5)	12.8	±	3.0	0.0	–	25.4	9(5.5)		0(0.0)		0(0.0)		0(0.0)
**(b)**																
**Market**	**Type**	**Samples,** **n**	**Positive Samples,** **n(%)**	**Concentration (** **μ** **g/L)**						**Concentration Distribution of Samples (** **μ** **g/kg/** **μ** **g/L), n (%)**						
				**Mean**	**±**	**SEM**	**Range**			**5–50**		**50–100**		**100–1000**		**>** **1000**
Shanghai	AQS	116	0(0.0)	0.0			0.0			0(0.0)		0(0.0)		0(0.0)		0(0.0)
	TRWS	83	7(8.4)	19.1	±	7.9	0.0	–	59.0	6(7.2)		1(1.2)		0(0.0)		0(0.0)
	TEWS	69	3(4.3)	13.3	±	4.4	4.6	–	18.1	3(4.3)		0(0.0)		0(0.0)		0(0.0)
Zhejiang	AQS	104	14(13.5)	7272.9	±	3312.0	1.1	–	36,090.3	2(1.9)		3(2.9)		3(2.9)		6(5.8)
	TRWS	85	5(5.9)	18.5	±	2.6	11.8	–	24.2	5(5.9)		0(0.0)		0(0.0)		0(0.0)
	TEWS	41	1(2.4)	13.2	±	0.0		–	13.2	1(2.4)		0(0.0)		0(0.0)		0(0.0)
Fujian	AQS	95	22(23.2)	3894.4	±	1592.1	0.4	–	30,593.6	4(4.2)		2(2.1)		8(8.4)		8(8.4)
	TRWS	79	15(19.0)	259.3	±	109.4	259.3	–	109.4	9(11.4)		1(1.3)		3(3.8)		2(2.5)
	TEWS	55	5(9.1)	12.4	±	5.2	0.0	–	25.4	5(9.1)		0(0.0)		0(0.0)		0(0.0)
Total	AQS	315	36(11.4)	5208.3	±	1609.8	0.4	–	36,090.3	6(1.9)		5(1.6)		11(3.5)		14(4.4)
	TRWS	247	27(10.9)	152.5	±	64.3	152.5	–	64.3	20(8.1)		2(0.8)		3(1.2)		2(0.8)
	TEWS	165	9(5.5)	12.8	±	3.0	0.0	–	25.4	9(5.5)		0(0.0)		0(0.0)		0(0.0)
**(c)**																
**Market**	**Type**	**Samples,** **n**	**Positive Samples,** **n (%)**	**Concentration (** **μ** **g/L)**						**Distribution in Different Months of a Year (** **μ** **g/kg/** **μ** **g/L), n (%)**						
				**Mean**	**±**	**SEM**	**Range**			**Apr.**	**May.**	**Jun.**	**Jul.**	**Aug.**	**Sep.**	**Oct.**
Eastern	AQS	279	32(11.5)	5470.9	±	1778.2	0.4	–	36,090.3	0(0.0)	2(0.7)	7(2.5)	2(0.7)	11(3.9)	4(1.4)	6(2.2)
	TRWS	231	26(11.3)	133.4	±	63.8	0.0	–	1366.3	2(0.9)	1(0.4)	2(0.9)	3(1.3)	12(5.2)	2(0.9)	4(1.7)
	TEWS	165	9(5.5)	12.8	±	3.0	0.0	–	25.4	0(0.0)	0(0.0)	6(3.6)	2(1.2)	1(0.6)	0(0.0)	0(0.0)
Southern	AQS	36	4(11.1)	3107.6	±	3011.4	53.1	–	12,141.6	1(2.8)	0(0.0)	1(2.8)	0(0.0)	0(0.0)	1(2.8)	1(2.8)
	TRWS	16	1(6.3)	648.0	±	0.0			648.0	0(0.0)	0(0.0)	0(0.0)	0(0.0)	0(0.0)	1(6.3)	0(0.0)
Total	AQS	315	36(11.4)	5208.3	±	1609.8	0.4	–	36,090.3	1(0.3)	2(0.6)	8(2.5)	2(0.6)	11(3.5)	5(1.6)	7(2.2)
	TRWS	247	27(10.9)	152.5	±	64.3	0.0	–	1366.3	2(0.8)	1(0.4)	2(0.8)	3(1.2)	12(4.9)	3(1.2)	4(1.6)
	TEWS	165	9(5.5)	12.8	±	3.0	0.0	–	25.4	0(0.0)	0(0.0)	6(3.6)	2(1.2)	1(0.6)	0(0.0)	0(0.0)

AQS: Aquatic Samples. TRWS: Transport Water Samples. TEWS: Temporary Water Samples.

## Data Availability

Data is contained within the article or supplementary material.

## Supplementary Materials

The following supporting information can be downloaded at https://www.mdpi.com/article/10.3390/foods13020238/s1.
