# Supplementary material for: Determination of Eugenol Residues in Fish Tissue, Transport, and Temporary Water of Aquatic Product by Gas Chromatography–Tandem Mass Spectrometry with Application of the Electrospun Nanofibrous Membrane
Source: Foods. 2024 Jan 11;13(2):238. doi: 10.3390/foods13020238 (PMC10814870; doi:10.3390/foods13020238)
Supplement: Supplementary file 1 [file foods-13-00238-s001.zip › foods-2780257-supplementary.pdf]

Table S1 The 2021 Aquatic Product Sampling Record Form

Sampling month	Name	Sampling amount	Base (pieces)	Place of origin	Sampling area	Sampling date
June	*Ophiocephalus argus*	1 piece	1000	Zhejiang	Shanghai	2021/6/16
	Mugil cephalus	1 piece	1000	Yancheng, Jiangsu	Shanghai	2021/6/16
	Oreochromis sp p	1 piece	1000	Xinghua, Jiangsu	Shanghai	2021/6/16
	Scophthalmus maximus	2 pieces	50	Shandong	Shanghai	2021/6/16
	Lateolabrax japonicus	1 piece	50	Jiangsu	Shanghai	2021/6/16
	Siniperca chuatsi	1 piece	50	Guangzhou	Shanghai	2021/6/16
	Acipensersinensi s	1 piece	50	Jiangsu	Shanghai	2021/6/16
	Epinephelussp	2 pieces	50	Fujian	Shanghai	2021/6/16
	Larimichthys crocea	1 piece	50	Xiangshan, Zhejiang	Shanghai	2021/6/16
	Oncorhynchus	1 block	10	Norway	Shanghai	2021/6/16
	Concha Ostreae	4 pieces	100	France	Shanghai	2021/6/16
	Pseudocardium sachalinense	4 pieces	200	Canada	Shanghai	2021/6/16
	Anoplopoma fimbria	1 piece	20	Norway	Shanghai	2021/6/16
	Cipangopaludia n cathayensis	0.5kg	1000	Hubei	Shanghai	2021/6/16
	Margarya francheti	0.6kg	2000	Jiangsu	Shanghai	2021/6/16
	Placopecta magellanicus	4 pieces	20	Dalian	Shanghai	2021/6/16
	Thunnus obesus	1 block	20	Norway	Shanghai	2021/6/16
July	*Siniperca chuatsi*	1 piece	20	Fujian	Fujian	2021/7/13
	Megalobrama amblycephala	2 pieces	200	Fujian	Fujian	2021/7/13
	Scophthalmus maximus	2 pieces	500	Shandong	Fujian	2021/7/13
	Lateolabrax japonicus	2 pieces	500	Fujian	Fujian	2021/7/13
	Pagrosomus major	2 pieces	200	Fujian	Fujian	2021/7/13
	Lateolabrax japonicus	2 pieces	100	Zhejiang	Zhejiang	2021/7/15
	Epinephelussp	2 pieces	200	Zhejiang	Zhejiang	2021/7/15

	Scophthalmus maximus	2 pieces	100	Shandong	Zhejiang	2021/7/15
	Pampus sinensis	2 pieces	50	Zhejiang	Zhejiang	2021/7/15
	Acipensersinensis	1 piece	20	Zhejiang	Zhejiang	2021/7/15
	Siniperca chuatsi	2 pieces	100	Jiangsu	Shanghai	2021/7/16
	Scophthalmus maximus	2 pieces	150	Shandong	Shanghai	2021/7/16
	Epinephelussp	2 pieces	100	Zhejiang	Shanghai	2021/7/16
	Carassius aumtus	3 pieces	1000	Jiangsu	Shanghai	2021/7/16
	Ophiocephalus argus	1 piece	1000	Shandong	Shanghai	2021/7/16
	Oncorhynchus	0.6kg	50kg	Norway	Fujian	2021/7/13
	Anoplopoma fimbria	1.1kg	50kg	America	Fujian	2021/7/13
	Placopecta magellanicus	1kg	50kg	Shandong	Fujian	2021/7/13
	Thunnus obesus	1kg	50kg	Canada	Fujian	2021/7/13
	Pseudocardium sachalinense	1kg	50kg	Canada	Fujian	2021/7/13
	Oncorhynchus	1kg	50kg	Norway	Shanghai	2021/7/16
	Concha Ostreae	6 pieces	200	Zhanjiang, Guangdong	Shanghai	2021/7/16
	Anoplopoma fimbria	1kg	50kg	America	Shanghai	2021/7/16
	Pseudocardium sachalinense	1kg	50kg	Canada	Shanghai	2021/7/16
August	*Thalassoma bifasciatum*	1 piece	20	Fujian	Fujian	2021/8/9
	Lateolabrax japonicus	2 pieces	200	Fujian	Fujian	2021/8/9
	Siniperca chuatsi	1 piece	50	Guangdong	Fujian	2021/8/9
	Pagrosomus major	2 pieces	50	Fujian	Fujian	2021/8/9
	Scophthalmus maximus	2 pieces	200	Shandong	Fujian	2021/8/9
	Acipensersinensis	2 pieces	100	Zhejiang	Zhejiang	2021/8/11
	Scophthalmus maximus	2 pieces	200	Shandong	Zhejiang	2021/8/11
	Pagrosomus major	2 pieces	100	Zhejiang	Zhejiang	2021/8/11

	Perca fluviatilis	2 pieces	50	Zhejiang	Zhejiang	2021/8/11
	Siniperca chuatsi	1 piece	20	Guangdong	Zhejiang	2021/8/11
	Epinephelussp	2 pieces	100	Guangdong	Shanghai	2021/8/12
	Siniperca chuatsi	2 pieces	150	Guangdong	Shanghai	2021/8/12
	Scophthalmus maximus	2 pieces	100	Shandong	Shanghai	2021/8/12
	Ophiocephalus argus	1 piece	1000	Anhui	Shanghai	2021/8/12
	Pagrosomus major	2 pieces	100	Zhejiang	Shanghai	2021/8/12
November	*Pampus sinensis*	2 pieces	20	Fujian	Shanghai	2021/11/6
	Siniperca chuatsi	2 pieces	200	Jiangsu	Shanghai	2021/11/6
	Pagrosomus major	2 pieces	50	Guangdong	Shanghai	2021/11/6
	Scophthalmus maximus	2 pieces	50	Shandong	Shanghai	2021/11/6
	Acipensersinensis	2 pieces	200	Jiangsu	Shanghai	2021/11/6

Table S2 The 2021 Transportation Water Sample Sampling Record Form

Sampling Month	Name	Sampling Amount	Base（ton）	Place of origin	Sampling area	Sampling date
June	*Ophiocephalus argus*	600mL	1	Zhejiang	Shanghai	2021/6/16
	Mugil cephalus	600mL	1	Jiangsu	Shanghai	2021/6/16
	Oreochromis spp	600mL	1	Jiangsu	Shanghai	2021/6/16
July	*Lateolabrax japonicus*	600mL	1	Fujian	Fujian	2021/7/13
	Penaeus chinensis	600mL	1	Fujian	Fujian	2021/7/13
	Scophthalmus maximus	600mL	1	Shandong	Fujian	2021/7/13
	Ophiocephalus argus	600mL	1	Jiangsu	Shanghai	2021/7/16
	Carassius aumtus	600mL	1	Shandong	Shanghai	2021/7/16
August	*Lateolabrax japonicus*	600mL	1	Fujian	Fujian	2021/8/9
	Thalassoma bifasciatum	600mL	1	Fujian	Fujian	2021/8/9
	Ophiocephalus argus	600mL	1	Anhui	Shanghai	2021/8/12
	Pseudobagrus fulvidraco	600mL	1	Jiangsu	Shanghai	2021/8/12
	Scophthalmus maximus	600mL	1	Shandong	Shanghai	2021/8/12
	Penaeus chinensis	601mL	1	Shanghai	Shanghai	2021/8/12
November	*Ophiocephalus argus*	600mL	1	Anhui	Shanghai	2021/11/6

	Epinephelussp	600mL	1	Jiangsu	Shanghai	2021/11/6
	Penaeus chinensis	600mL	1	Shanghai	Shanghai	2021/11/6

Table S3 The 2021 Temporary Water Samples Sampling Record Form

Sampling month	Name	Sampling amount (mL)	Base (ton)	Place of origin	Sampling area	Sampling date
June	*Scophthalmus maximus* *Lateolabrax japonicus* *Siniperca chuatsi*	600	1	Shanghai	Shanghai	2021/6/16
	Acipensersinensis *Epinephelussp*	600	1	Shanghai	Shanghai	2021/6/16
July	*Pagrosomus major*	600	1	Fujian	Fujian	2021/7/13
	Lateolabrax japonicus *Epinephelussp* *Scophthalmus maximus* *Pampus sinensis* *Acipensersinensis*	600	1	Zhejiang	Zhejiang	2021/7/15
	Scophthalmus maximus *Epinephelussp*	600	1	Shanghai	Shanghai	2021/7/16
	Siniperca chuatsi	600	1	Shanghai	Shanghai	2021/7/16
August	*Scophthalmus maximus*	600	1	Fujian	Fujian	2021/8/9
	Siniperca chuatsi	600	2	Fujian	Fujian	2021/8/9
	Lateolabrax japonicus, *Acipensersinensis* *Scophthalmus maximus* *Pagrosomus major*	600	2	Zhejiang	Zhejiang	2021/8/11
	Siniperca chuatsi	600	2	Zhejiang	Zhejiang	2021/8/11
	Epinephelussp	600	2	Shanghai	Shanghai	2021/8/12
	Siniperca chuatsi *Scophthalmus maximus*	600	2	Shanghai	Shanghai	2021/8/12

	Siniperca chuatsi	600	2	Shanghai	Shanghai	2021/8/12
November	*Siniperca chuatsi*	600	1	Shanghai	Shanghai	2021/11/6
	Pagrosomus major	600	1	Shanghai	Shanghai	2021/11/6

Table S4 Sampling Record Table for April 2022

Name	Sampling amount	Base (pieces)	Weight （kg）	Place of origin	Sampling area	Sampling date
Erythroculter ilishaeformis	2	200	1.5	Shanghai	Shanghai	2022/4/15
Lateolabrax japonicus	2	400	2	Shanghai	Shanghai	2022/4/15
Pseudobagrus fulvidraco	5	800	2.1	Zhejiang	Shanghai	2022/4/15
Ophiocephalus argus	1	300	0.9	Shanghai	Shanghai	2022/4/15
Carassius aumtus	2	500	3	Shanghai	Shanghai	2022/4/15
Scophthalmus maximus	2	200	2.5	Shandong	Shanghai	2022/4/15
Leiocassis longirostris	2	200	1.7	Jiangsu	Shanghai	2022/4/15
Oreochromis spp	2	100	2.2	Jiangsu	Shanghai	2022/4/15
Lateolabrax japonicus	1	200	2.5	Shanghai	Shanghai	2022/4/15
Siniperca chuatsi	2	200	2.9	Guangzhou	Shanghai	2022/4/15
Ophiocephalus argus	2	400	3.3	Anhui	Shanghai	2022/4/15
Carassius aumtus	6	1000	4	Dianshan Lake,Shanghai	Shanghai	2022/4/16
Hypophthalmichthys molitrix	2	500	5	Shanghai	Shanghai	2022/4/16
Carassius aumtus	4	1000	3.5	Chongming Island, Shanghai	Shanghai	2022/4/16
Ctenopharyngodon idellus	2	300	4.5	Sheyang, Jiangsu	Shanghai	2022/4/16
Pampus sinensis	2	100	2.5	Fujian	Fujian	2022/4/16
Scophthalmus maximus	2	200	3.0	Shandong	Fujian	2022/4/16
Epinephelussp	2	200	3.1	Fujian	Fujian	2022/4/16
Siniperca chuatsi	2	100	3.5	Guangdong	Fujian	2022/4/16
Pseudobagrus fulvidraco	4	200	2.5	Fujian	Fujian	2022/4/16

Lateolabrax japonicus	2	100	2	Guangdong	Fujian	2022/4/16
Ophiocephalus argus	1	50	1.8	Jiangxi	Fujian	2022/4/16
Siniperca chuatsi	2	200	2.6	Guangdong	Fujian	2022/4/16
Scophthalmus maximus	2	200	3	Fujian	Fujian	2022/4/16
Epinephelussp	2	200	2.1	Hainan	Fujian	2022/4/16
Pagrosomus major	2	50	2	Ningde,Fujian	Fujian	2022/4/16
Megalobrama amblycephala	2	100	2.5	Fujian	Fujian	2022/4/16
Pseudobagrus fulvidraco	4	50	2.2	Fujian	Fujian	2022/4/16
Ctenopharyngodon idellus	1	100	2	Fujian	Fujian	2022/4/16
Carassius aumtus	4	500	2.3	Jiangsu	Fujian	2022/4/16
Hypophthalmichthys nobilis	1		2.4	Huzhou	Zhejiang	2022/4/16
Siniperca chuatsi	2		1.3	Fujian	Zhejiang	2022/4/16
Carassius aumtus	2		1.5	Huzhou	Zhejiang	2022/4/16
Megalobrama amblycephala	2		1.4	Huzhou	Zhejiang	2022/4/16
Lateolabrax japonicus	2		1.25	Huzhou	Zhejiang	2022/4/16
Scophthalmus maximus	2		1.1	Shandong	Zhejiang	2022/4/16
Pagrosomus major	2		0.85	Fujian	Zhejiang	2022/4/16
Lateolabrax japonicus	2		1.3	Fujian	Zhejiang	2022/4/16
Scophthalmus maximus	2		1.35	Shandong	Zhejiang	2022/4/16
Ophiocephalus argus	6		2.2	Guangdong	Zhejiang	2022/4/16
Hypophthalmichthys nobilis	2		7	Huzhou	Zhejiang	2022/4/16
Carassius aumtus	12		1.35	Huzhou	Zhejiang	2022/4/16
Carassius aumtus	8		0.3	Huzhou	Zhejiang	2022/4/16
Ophiocephalus argus	2		0.8	Guangdong	Zhejiang	2022/4/17
Lateolabrax japonicus	2		1.5	Guangzhou	Zhejiang	2022/4/17
Cyprinus carpio	2		5	Qiandao Lake	Zhejiang	2022/4/17
Megalobrama amblycephala	2		6	Qiandao Lake	Zhejiang	2022/4/17

Table S5 Sampling Record Table for May 2022

Name	Amount	Base（pieces）	Weight（kg）	Place of origin	Sampling area	Sampling date
Pseudobagrus fulvidraco	8	1000	2.05	Shanghai	Shanghai	2022/5/18
Mugil cephalus	2	50	2.09	Shanghai	Shanghai	2022/5/18
Ophiocephalus argus	2	100	2	Zhejiang	Shanghai	2022/5/18
Penaeus monodon	30	1000	1.05	Shanghai	Shanghai	2022/5/18
Scophthalmus maximus	3	50	2.15	Shandong	Shanghai	2022/5/18
Siniperca chuatsi	2	100	1.9	Guangzhou	Shanghai	2022/5/18
Lateolabrax japonicus	2	100	2.04	Guangzhou	Shanghai	2022/5/18
Epinephelussp	2	100	1.65	Fujian	Shanghai	2022/5/18
Ietalurus Punetaus	2	100	2	Jiangsu	Shanghai	2022/5/18
Megalobrama amblycephala	2	100	2.01	Shanghai	Shanghai	2022/5/18
Ctenopharyngodon idellus	2	100	1.9	Shanghai	Shanghai	2022/5/19
Hypophthalmichthys nobilis	2	100	2.25	Shanghai	Shanghai	2022/5/19
Pseudobagrus fulvidraco	8	1000	2.3	Shanghai	Shanghai	2022/5/19
Lateolabrax japonicus	2	100	2.1	Guangzhou	Shanghai	2022/5/19
Ophiocephalus argus	2	50	2.3	Shanghai	Shanghai	2022/5/19
Carassius aumtus	2	1000	1.5	Jiangsu	Zhejiang	2022/5/18
Megalobrama amblycephala	1	2000	2	Jiangxi	Zhejiang	2022/5/18
Ctenopharyngodon idellus	1	300	3	Jiangsu	Zhejiang	2022/5/18
Lateolabrax japonicus	2	200	2.5	Zhejiang	Zhejiang	2022/5/18
Carassius aumtus	2	3000	1.5	Jiangsu	Zhejiang	2022/5/18
Hypophthalmichthys nobilis	1	400	3	Anhui	Zhejiang	2022/5/18
Megalobrama amblycephala	1	1000	2	Zhejiang	Zhejiang	2022/5/18
Ophiocephalus argus	1	300	2	Shanghai	Zhejiang	2022/5/18
Ophiocephalus argus	1	100	3	Zhejiang	Zhejiang	2022/5/18
Carassius aumtus	2	3000	2	Jiangsu	Zhejiang	2022/5/18
Ophiocephalus argus	1	100	2	Zhejiang	Zhejiang	2022/5/18
Pseudobagrus	7	1000	3	Anhui	Zhejiang	2022/5/19

fulvidraco						
Ophiocephalus argus	1	2000	2	Anhui	Zhejiang	2022/5/19
Oreochromis spp	1	1000	2	Zhejiang	Zhejiang	2022/5/19
Megalobrama amblycephala	1	20	1.5	Zhejiang	Zhejiang	2022/5/19
Lateolabrax japonicus	2	1000	0.85	Fujian	Fujian	2022/5/18
Pampus sinensis	3	500	1.55	Fujian	Fujian	2022/5/18
Pagrosomus major	4	2000	1.36	Fujian	Fujian	2022/5/18
Penaeus vannamei	50	10000	1.25	Fujian	Fujian	2022/5/18
Larimichthys crocea	1	200	1.45	Ningde	Fujian	2022/5/18
Larimichthys crocea	2	1000	1.68	Ningde	Fujian	2022/5/19
Scophthalmus maximus	2	200	1.44	Shandong	Fujian	2022/5/19
Megalobrama amblycephala	2	2000	1.31	Fujian	Fujian	2022/5/19
Lateolabrax japonicus	2	1000	0.85	Fujian	Fujian	2022/5/19
Siniperca chuatsi	2	500	1.35	Fujian	Fujian	2022/5/19
Pseudobagrus fulvidraco	4	2000	1.05	Fujian	Fujian	2022/5/19
Hypophthalmichthys nobilis	2	2000	2.5	Fujian	Fujian	2022/5/20
Hypophthalmichthys molitrix	2	2000	2.3	Fujian	Fujian	2022/5/20
Ctenopharyngodon idellus	2	1500	2.4	Fujian	Fujian	2022/5/20
Carassius aumtus	4	5000	1.3	Fujian	Fujian	2022/5/20

Table S6 Sampling Record Table for May 2022

Name	Amount	Base （pieces）	Weight （kg）	Place of origin	Sampling area	Sampling date
Hypophthalmichthys molitrix	1	1000	1.5	Jiangsu	Shanghai	2022/6/17
opharyngodon idellus	1	500	3	Jiangsu	Shanghai	2022/6/17
Hypophthalmichthys nobilis	1	2000	3.5	Qiandao Lake	Shanghai	2022/6/17
Carassius aumtus	2	2000	0.75	Qiandao Lake	Shanghai	2022/6/17
Ophiocephalus argus	2	1000	0.8	Guangdong	Shanghai	2022/6/17
Scophthalmus maximus	2	5000	1.15	Shandong	Shanghai	2022/6/18
Siniperca chuatsi	2	500	1.35	Zhejiang	Shanghai	2022/6/18
Pseudobagrus fulvidraco	4	2000	0.65	Shanghai	Shanghai	2022/6/18

Mugil cephalus	3	1000	1.5	Zhejiang	Shanghai	2022/6/18
Ophiocephalus argus	2	500	2.4	Zhejiang	Shanghai	2022/6/18
Pampus sinensis	2	200	1.35	Fujian	Shanghai	2022/6/18
Epinephelussp	2	200	1.35	Fujian	Shanghai	2022/6/18
Carassius aumtus	2	200	1.5	Shanghai	Shanghai	2022/6/18
Megalobrama amblycephala	2	200	1.5	Qianddao Lake	Shanghai	2022/6/18
Lateolabrax japonicus	2	5000	1.2	Sanghai	Shanghai	2022/6/18
Ctenopharyngodon idellus	1	800	2	Jiangsu	Zhejiang	2015/6/17
Hypophthalmichthys nobilis	1	2000	1.9	Jiangsu	Zhejiang	2015/6/17
Hypophthalmichthys nobilis	1	1000	2.6	Jiangxi	Zhejiang	2015/6/17
Scophthalmus maximus	2	2000	3	Jiangsu	Zhejiang	2022/6/18
Siniperca chuatsi	1	200	1.5	Zhejiang	Zhejiang	2022/6/18
Lateolabrax japonicus	1	500	1.6	Zhejiang	Zhejiang	2022/6/18
Carassius aumtus	2	1000	2.1	Zhejiang	Zhejiang	2022/6/18
Ctenopharyngodon idellus	1	300	3	Zhejiang	Zhejiang	2022/6/18
Ophiocephalus argus	1	200	4	Zhejiang	Zhejiang	2022/6/18
Hypophthalmichthys nobilis	1	1000	1.9	Jiangxi	Zhejiang	2022/6/18
Ophiocephalus argus	1	1000	2.6	Zhejiang	Zhejiang	2022/6/18
SciaemopsOcellatus	2	2000	1.7	Fujian	Zhejiang	2022/6/19
Siniperca chuatsi	1	1000	2.2	Zhejiang	Zhejiang	2022/6/19
Pseudobagrus fulvidraco	8	4000	2.5	Zhejiang	Zhejiang	2022/6/19
Larimichthys crocea	1	800	1.8	Fujian	Zhejiang	2022/6/19
Scophthalmus maximus	3	1000	1.5	Shandong	Fujian	2022/6/16
Mugil cephalus	2	200	2.8	Fujian	Fujian	2022/6/16
Ophiocephalus argus	2	2000	3.1	Foshan	Fujian	2022/6/16
Siniperca chuatsi	2	1000	2.9	Fujian	Fujian	2022/6/16
Lateolabrax japonicus	3	500	2.8	Fujian	Fujian	2022/6/16
Megalobrama amblycephala	1	2000	2.4	Fujian	Fujian	2022/6/16
Carassius aumtus	3	2000	2	Fujian	Fujian	2022/6/16
Siniperca chuatsi	2	1000	2.4	Guangzhou	Fujian	2022/6/17

Pseudobagrus fulvidraco	4	500	1.9	Guangzhou	Fujian	2022/6/17
Lateolabrax japonicus	3	2000	2.1	Guangzhou	Fujian	2022/6/17
Megalobrama amblycephala	2	10000	2.9	Guangzhou	Fujian	2022/6/17
Scophthalmus maximus	1	200	1.1	Xiamen	Fujian	2022/6/17
Cyprinus carpio	3	400	1.4	Shandong	Fujian	2022/6/17
Leiocassis longirostris	1	1000	2	Shandong	Fujian	2022/6/17
Acipensersinensis	1	500	1.5	Nanning	Fujian	2022/6/17

Table S7 Sampling Record Table for July 2022

Name	Amount	Base	Weight（kg）	Place of origin	Sampling area	Sampling date
Scophthalmus maximus	2	500	1.35	Shandong	Shanghai	2022/7/13
Epinephelussp	2	200	1.25	Guangdong	Shanghai	2022/7/13
Megalobrama amblycephala	2	200	1.9	Suzhou	Shanghai	2022/7/13
Siniperca chuatsi	2	500	1.25	Fujian	Shanghai	2022/7/13
Ophiocephalus argus	1	1000	2.4	Guangdong	Shanghai	2022/7/13
Mugil cephalus	3	2000	2.1	Zhejiang	Shanghai	2022/7/13
Carassius aumtus	2	300	1.35	Qiandao Lake	Shanghai	2022/7/13
Hypophthalmichthys nobilis	1	200	2.5	Qiandao Lake	Shanghai	2022/7/13
Pagrosomus major	2	200	1.4	Fujian	Shanghai	2022/7/13
Pampus sinensis	2	100	1.1	Zhejiang	Shanghai	2022/7/13
Hypophthalmichthys nobilis	1	100	2.2	Qiandao Lake	Shanghai	2022/7/14
Ctenopharyngodon idellus	1	100	1.5	Jiangsu	Shanghai	2022/7/14
Carassius aumtus	4	500	1.5	Shanghai	Shanghai	2022/7/14
Siniperca chuatsi	2	200	1.5	Jiangsu	Shanghai	2022/7/14
Lateolabrax japonicus	2	200	1.1	Zhejiang	Shanghai	2022/7/14
Carassius aumtus	2	100pieces	1.2	Jiangsu	Zhejiang	2022/7/13
Siniperca chuatsi	1	100pieces	0.9	Guangdong	Zhejiang	2022/7/13
Ophiocephalus argus	1	200pieces	1.5	Jiangsu	Zhejiang	2022/7/13
Lateolabrax japonicus	2	200pieces	1.2	Zhejiang	Zhejiang	2022/7/13
Mugil cephalus	2	100pieces	1	Zhejiang	Zhejiang	2022/7/13

Hypophthalmichthys nobilis	1	100pieces	1.6	Zhejiang	Zhejiang	2022/7/13
Pseudobagrus fulvidraco	4	100pieces	1.3	Jiangsu	Zhejiang	2022/7/13
Siniperca chuatsi	1	50pieces	0.8	Guangdong	Zhejiang	2022/7/14
Epinephelussp	2	100pieces	1.1	Guangdong	Zhejiang	2022/7/14
Megalobrama amblycephala	1	50pieces	1.2	Jiangsu	Zhejiang	2022/7/14
Carassius aumtus	2	100pieces	1.2	Zhejiang	Zhejiang	2022/7/14
Scophthalmus maximus	2	100pieces	1.4	Fujian	Zhejiang	2022/7/14
Lateolabrax japonicus	2	100pieces	1.5	Zhejiang	Zhejiang	2022/7/14
Lateolabrax japonicus	2	100pieces	1.4	Zhejiang	Zhejiang	2022/7/14
Pagrosomus major	2	100pieces	1.3	Fujian	Zhejiang	2022/7/14
Epinephelussp	1	1000	1.5	Fujian	Fujian	2022/7/9
Pagrosomus major	3	2000	1.9	Fujian	Fujian	2022/7/9
Epinephelussp	1	20	1.3	Fujian	Fujian	2022/7/9
Larimichthys crocea	2	400	2.2	Fujian	Fujian	2022/7/9
Scophthalmus maximus	2	3000	2.5	Shandong	Fujian	2022/7/9
Lateolabrax japonicus	2	1000	0.9	Fujian	Fujian	2022/7/9
Epinephelussp	1	800	1.2	Fujian	Fujian	2022/7/10
Scophthalmus maximus	2	400	1.5	Fujian	Fujian	2022/7/10
Scophthalmus maximus	2	3000	1.6	Shandong	Fujian	2022/7/10
Scophthalmus maximus	2	2000	1.3	Shandong	Fujian	2022/7/10
Megalobrama amblycephala	2	1000	1.3	Fujian	Fujian	2022/7/10

Table S8 Sampling Record Table for August 2022

Name	Amount	Base	Weight （kg）	Place of origin	Sampling area	Sampling date
Lateolabrax japonicus	2	2000	0.9	Shanghai	Shanghai	2022/8/19
Megalobrama amblycephala	1	1000	1.2	Jiangsu	Shanghai	2022/8/19
Carassius aumtus	2	2000	1.1	Zhejiang	Shanghai	2022/8/19

Hypophthalmichthys nobilis	1	2000	1.5	Jiangsu	Shanghai	2022/8/19
Ophiocephalus argus	1	2000	0.8	Shandong	Shanghai	2022/8/19
Pseudobagrus fulvidraco	4	2000	1.3	Anhui	Shanghai	2022/8/19
Pseudobagrus fulvidraco	4	2000	1.2	Jiangsu	Shanghai	2022/8/19
Siniperca chuatsi	2	2000	1	Guangdong	Shanghai	2022/8/20
Lateolabrax japonicus	2	1	1.2	Shanghai	Shanghai	2022/8/20
Scophthalmus maximus	2	1	1	Fujian	Shanghai	2022/8/20
Larimichthys crocea	3	1000	1.5	Fujian	Zhejiang	2022/8/19
Carassius aumtus	3	2000	0.9	Jiangsu	Zhejiang	2022/8/19
Larimichthys crocea	2	200	1.2	Fujian	Zhejiang	2022/8/19
Ctenopharyngodon idellus	1	200	1.5	Zhejiang	Zhejiang	2022/8/19
Lateolabrax japonicus	3	4000	1.7	Zhejiang	Zhejiang	2022/8/20
Scophthalmus maximus	2	200	0.8	Shandong	Zhejiang	2022/8/20
Ctenopharyngodon idellus	2	400	2	Jiangxi	Zhejiang	2022/8/20
Hypophthalmichthys nobilis	1	2000	1.4	Jiangxi	Zhejiang	2022/8/20
Hypophthalmichthys nobilis	1	2000	1.8	Jiangsu	Zhejiang	2022/8/20
Ophiocephalus argus	1	3000	2.1	Zhejiang	Zhejiang	2022/8/20
Ophiocephalus argus	1	1000	1.8	Zhejiang	Zhejiang	2022/8/20
Epinephelussp	2	3000	1.3	Shandong	Zhejiang	2022/8/21
Epinephelussp	2	1000	1.3	Fujian	Fujian	2022/8/18
Penaeus monodon	50	20000	1.25	Fujian	Fujian	2022/8/18
Carassius aumtus	8	5000	1.9	Fujian	Fujian	2022/8/19
Hypophthalmichthys nobilis	2	1000	1.25	Fujian	Fujian	2022/8/19
Ctenopharyngodon idellus	2	1000	2.4	Fujian	Fujian	2022/8/19
Epinephelussp	2	1000	1.45	Fujian	Fujian	2022/8/20
Sciaenops ocellatus	2	500	2.05	Fujian	Fujian	2022/8/20
Pagrosomus major	2	500	2.5	Fujian	Fujian	2022/8/20
Pampus sinensis	2	500	1.2	Fujian	Fujian	2022/8/20
Lateolabrax japonicus	2	1000	2.2	Fujian	Fujian	2022/8/20

Table S9 Sampling Record Table for September 2022

Name	Amount	Base	Weight （kg）	Place of origin	Sampling area	Sampling date
Carassius aumtus	12	2000	3.2	Shanghai	Shanghai	2022/9/23
Pseudobagrus fulvidraco	12	2000	2.7	Shanghai	Shanghai	2022/9/23
Lateolabrax japonicus	4	2000	2.5	Shanghai	Shanghai	2022/9/23
Pseudobagrus fulvidraco	13	2000	2.6	Jiangsu	Shanghai	2022/9/23
Pseudobagrus fulvidraco	12	2000	2.8	Zhejiang	Shanghai	2022/9/23
Erythroculter ilishaeformis	4	2000	2.9	Zhejiang	Shanghai	2022/9/23
Penaeus vannamei Boone	30	20000	1.8	Jiangsu	Shanghai	2022/9/23
Penaeus monodon	40	20000	2.1	Jiangsu	Shanghai	2022/9/23
Ietalurus Punetaus	3	1000	3.5	Anhui	Shanghai	2022/9/23
Oxyeleotris marmorata	2	500	1.3	Guangdong	Shanghai	2022/9/23
Ctenopharyngodon idellus	2	1000	2.5	Jiangsu	Zhejiang	2022/9/23
Carassius aumtus	6	2000	2.5	Zhejiang	Zhejiang	2022/9/23
Hypophthalmichthys nobilis	2	500	1.3	Zhejiang	Zhejiang	2022/9/23
Lateolabrax japonicus	2	500	1.4	Jiangsu	Zhejiang	2022/9/23
Pagrosomus major	2	500	1.1	Zhejiang	Zhejiang	2022/9/23
Lateolabrax japonicus	2	1000	1.5	Zhejiang	Zhejiang	2022/9/24
Siniperca chuatsi	2	500	1.45	Zhejiang	Zhejiang	2022/9/24
Ctenopharyngodon idellus	2	200	2	Jiangsu	Zhejiang	2022/9/24
Hypophthalmichthys nobilis	2	100	2.5	Zhejiang	Zhejiang	2022/9/24
Mylopharyngdon piceus Richardson	2	200	2	Jiangsu	Zhejiang	2022/9/24
Pseudobagrus fulvidraco	5	10000	1.6	Fujian	Fujian	2022/9/23
Carassius aumtus	2	10000	1.2	Fujian	Fujian	2022/9/23
Siniperca chuatsi	2	5000	1.1	Fujian	Fujian	2022/9/23
Tinca tinca	2	10000	1.6	Fujian	Fujian	2022/9/23
Scophthalmus maximus	2	10000	1.4	Fujian	Fujian	2022/9/23

Siniperca chuatsi	2	5000	1.2	Fujian	Fujian	2022/9/24
Scophthalmus maximus	2	10000	1.2	Shandong	Fujian	2022/9/24
Trachinotus ovatus	2	10000	1.8	Fujian	Fujian	2022/9/24
Epinephelussp	2	5000	1.1	Fujian	Fujian	2022/9/24
Lateolabrax japonicus	2	10000	1.4	Guangdong	Fujian	2022/9/24

Table S10 Sampling Record Table for October 2022

Name	Amount	Base	Weight（kg）	Place of origin	Sampling area	Sampling date
Macrobra'chiumrosen bergiideman	12	2000	2.1	Shanghai	Shanghai	2022/10/22
Pseudobagrus fulvidraco	12	2000	3	Zhejiang	Shanghai	2022/10/22
Penaeus monodon	4	2000	1.6	Shanghai	Shanghai	2022/10/22
Pseudobagrus fulvidraco	13	2000	3.1	Jiangsu	Shanghai	2022/10/22
Lateolabrax japonicus	12	2000	3.1	Guangdong	Shanghai	2022/10/22
Ophiocephalus argus	4	2000	4.1	Zhejiang	Shanghai	2022/10/22
Carassius aumtus	30	20000	3.2	Jiangsu	Shanghai	2022/10/23
Penaeus vannamei Boone	40	20000	3.1	Zhejiang	Shanghai	2022/10/23
Cyprinus carpio	3	1000	4.2	Jiangsu	Shanghai	2022/10/23
Carassius aumtus	2	500	3.5	Jiangsu	Shanghai	2022/10/23
Ophiocephalus argus	1	10000	1.6	Shandong	Zhejiang	2022/10/26
Lateolabrax japonicus	2	10000	1.2	Zhejiang	Zhejiang	2022/10/26
Pseudobagrus fulvidraco	5	5000	1.1	Anhui	Zhejiang	2022/10/26
Siniperca chuatsi	2	10000	1.6	Guangdong	Zhejiang	2022/10/26
Carassius aumtus	2	10000	1.4	Zhejiang	Zhejiang	2022/10/26
Siniperca chuatsi	2	5000	1.2	Guangdong	Zhejiang	2022/10/26
Lateolabrax japonicus	2	10000	1.2	Zhejiang	Zhejiang	2022/10/26
Pagrosomus major	2	10000	1.8	Fujian	Zhejiang	2022/10/26
Scophthalmus maximus	2	5000	1.1	Fujian	Zhejiang	2022/10/26
Hypoplectrus indigo	2	10000	1.4	Guangdong	Zhejiang	2022/10/26
Epinephelussp	2	300	1.3	Shanghai	Fujian	2022/10/26
Sciaenops ocellatus	2	500	2	Jiangsu	Fujian	2022/10/26

Ctenopharyngodon idellus	4	1000	3.3	Zhejiang	Fujian	2022/10/27
Hypophthalmichthys nobilis	4	1000	3.5	Jiangsu	Fujian	2022/10/27
Carassius aumtus	10	5000	2.9	Shandong	Fujian	2022/10/27
Trachinotus ovatus	2	500	1.2	Anhui	Fujian	2022/10/28
Lateolabrax japonicus	2	500	1.6	Jiangsu	Fujian	2022/10/28
Epinephelussp	2	1000	1.5	Guangdong	Fujian	2022/10/28
Pagrosomus major	2	500	1.5	Shanghai	Fujian	2022/10/28
Sciaenops ocellatus	2	500	1.7	Fujian	Fujian	2022/10/28

Table S11 The 2022 Transportation Water Samples Sampling Record Form

**Sampling month**	***Name***	**Sampling amount**	**Base**	**Place of origin**	**Sampling area**	**Sampling date**
	Erythroculter lishaeformis	550mL	1ton	Shanghai	Shanghai	2022/4/15
	Lateolabrax japonicus	550mL	1ton	Shanghai	Shanghai	2022/4/15
	Pseudobagrus fulvidraco	550mL	1ton	Zhejiang	Shanghai	2022/4/15
	Ophiocephalus argus	550mL	1ton	Shanghai	Shanghai	2022/4/15
	Carassius aumtus	550mL	1ton	Shanghai	Shanghai	2022/4/15
	Scophthalmus maximus	550mL	1ton	Shandong	Shanghai	2022/4/15
	Leiocassis longirostris	550mL	1ton	Jiangsu	Shanghai	2022/4/15
	Oreochromisspp	550mL	1ton	Jiangsu	Shanghai	2022/4/15
	Lateolabrax japonicus	550mL	1ton	Shanghai	Shanghai	2022/4/15
	Siniperca chuatsi	550mL	1ton	Guangzhou	Shanghai	2022/4/15
	Ophiocephalus argus	550mL	1ton	Anhui	Shanghai	2022/4/15
	Carassius aumtus	550mL	1ton	Dianshan Lake,Shanghai	Shanghai	2022/4/16
April	*Hypophthalmichthys molitrix*	550mL	1ton	Shanghai	Shanghai	2022/4/16
	Carassius aumtus	550mL	1ton	Chongming Island,Shanghai	Shanghai	2022/4/16
	Ctenopharyngodon idellus	550mL	1ton	Sheyang,Jiangsu	Shanghai	2022/4/16
	Lateolabrax japonicus	350mL	1ton	Fujian	Fujian	2022/4/14
	Pampus sinensis	350mL	1ton	Fujian	Fujian	2022/4/14
	Epinephelussp	350mL	1ton	Fujian	Fujian	2022/4/14
	Lateolabrax japonicus	350mL	1ton	Fujian	Fujian	2022/4/14
	Scophthalmus maximus	350mL	1ton	Fujian	Fujian	2022/4/14
	Epinephelus tauvina	350mL	1ton	Fujian	Fujian	2022/4/14
	Scophthalmus maximus	350mL	1ton	Fujian	Fujian	2022/4/14
	Scophthalmus maximus	350mL	1ton	Shandong	Fujian	2022/4/14
	Epinephelussp	350mL	1ton	Fujian	Fujian	2022/4/16
	Penaeus monodon Fabricius	350mL	1ton	Fujian	Fujian	2022/4/16

	Pseudobagrus fulvidraco	350mL	1ton	Fujian	Fujian	2022/4/16
	Siniperca chuats	350mL	1ton	Fujian	Fujian	2022/4/16
	Ctenopharyngodon idellus	350mL	1ton	Fujian	Fujian	2022/4/16
	Hypophthalmichthys nobilis	350mL	1ton	Fujian	Fujian	2022/4/16
	Hypophthalmichthys molitrix	350mL	1ton	Fujian	Fujian	2022/4/16
May	*Penaeus monodon*	550mL	5ton	Shanghai	Shanghai	2022/5/18
	Ophiocephalus argus	550mL	5ton	Zhejiang	Shanghai	2022/5/18
	Scophthalmus maximus	550mL	5ton	Shandong	Shanghai	2022/5/18
	Siniperca chuatsi	550mL	5ton	Guangzhou	Shanghai	2022/5/18
	Mugil cephalus	550mL	5ton	Shanghai	Shanghai	2022/5/18
	Pseudobagrus fulvidraco	550mL	5ton	Shanghai	Shanghai	2022/5/18
	Carassius aumtus	550mL	20ton	Jiangsu	Zhejiang	2022/5/18
	Megalobrama amblycephala	550mL	30ton	Jiangxi	Zhejiang	2022/5/18
	Ctenopharyngodon idellus	550mL	25ton	Jiangsu	Zhejiang	2022/5/18
	Lateolabrax japonicus	550mL	10ton	Zhejiang	Zhejiang	2022/5/18
	Carassius aumtus	550mL	20ton	Jiangsu	Zhejiang	2022/5/18
	Hypophthalmichthys nobilis	550mL	25ton	Anhui	Zhejiang	2022/5/18
	Megalobrama amblycephala	550mL	20ton	Zhejiang	Zhejiang	2022/5/18
	Ophiocephalus argus	550mL	20ton	Shanghai	Zhejiang	2022/5/18
	Ophiocephalus argus	550mL	20ton	Zhejiang	Zhejiang	2022/5/18
	Carassius aumtus	550mL	25ton	Jiangsu	Zhejiang	2022/5/18
	Ophiocephalus argus	550mL	20ton	Zhejiang	Zhejiang	2022/5/18
	Pseudobagrus fulvidraco	550mL	30ton	Anhui	Zhejiang	2022/5/19
	Ophiocephalus argus	550mL	20ton	Anhui	Zhejiang	2022/5/19
	Oreochromis pp	550mL	30ton	Zhejiang	Zhejiang	2022/5/19
	Megalobrama amblycephala	550mL	20ton	Zhejiang	Zhejiang	2022/5/19
	Lateolabrax japonicus （bottle×1）	550mL	1ton	Xiamen	Fujian	2022/5/18
	Pampus sinensis （bottle×3）	550mL	1ton	Xiamen	Fujian	2022/5/18
	Pagrosomus major （bottle×5）	550mL	1ton	Xiamen	Fujian	2022/5/18
	Penaeus vannamei （×7）	550mL	1ton	Xiamen	Fujian	2022/5/18
	Larimichthys crocea 105 （bottle×9）	550mL	1ton	Xiamen	Fujian	2022/5/18
	Larimichthys crocea （bottle F1）	550mL	2ton	Fuzhou	Fujian	2022/5/19
	Megalobrama amblycephala (bottle F4)	550mL	1ton	Fuzhou	Fujian	2022/5/19
	Lateolabrax japonicus （bottle F6）	550mL	1ton	Fuzhou	Fujian	2022/5/19
	Pseudobagrus fulvidraco （bottle F9）	550mL	1ton	Fuzhou	Fujian	2022/5/19
	Hypophthalmichthys nobilis （bottle F11）	550mL	1ton	Fuzhou	Fujian	2022/5/20
	Hypophthalmichthys molitrix	550mL	1ton	Fuzhou	Fujian	2022/5/20

	(bottle F13)					
	Ctenopharyngodon idellus (bottle F15)	550mL	1ton	Fuzhou	Fujian	2022/5/20
	Carassius aumtus (bottle F7)	550mL	1ton	Fuzhou	Fujian	2022/5/20
June	*Hypophthalmichthys molitrix*	550mL	1000L	Shanghai	Shanghai	2022/6/17
	Ctenopharyngodon idellus	550mL	1000L	Shanghai	Shanghai	2022/6/17
	Hypophthalmichthys nobilis	550mL	1000L	Shanghai	Shanghai	2022/6/17
	Carassius aumtus	550mL	1000L	Shanghai	Shanghai	2022/6/17
	Scophthalmus maximus	550mL	2000L	Shanghai	Shanghai	2022/6/18
	Siniperca chuatsi	550mL	2000L	Shanghai	Shanghai	2022/6/18
	Mugil cephalus	550mL	2000L	Shanghai	Shanghai	2022/6/18
	Ophiocephalus argus	550mL	2000L	Shanghai	Shanghai	2022/6/18
	Pampus sinensis	550mL	1000L	Shanghai	Shanghai	2022/6/18
	Carassius aumtus	550mL	2000L	Shanghai	Shanghai	2022/6/18
	Megalobrama amblycephala	550mL	2000L	Shanghai	Shanghai	2022/6/18
	Ctenopharyngodon idellus	550mL	1ton	Jiangsu	Zhejiang	2022/6/17
	Ctenopharyngodon idellus	550mL	1ton	Jiangsu	Zhejiang	2022/6/17
	Hypophthalmichthys nobilis	550mL	1ton	Jiangxi	Zhejiang	2022/6/17
	Scophthalmus maximus	550mL	1ton	Jiangsu	Zhejiang	2022/6/18
	Siniperca chuatsi	550mL	1ton	Zhejiang	Zhejiang	2022/6/18
	Lateolabrax japonicas	550mL	1ton	Zhejiang	Zhejiang	2022/6/18
	Carassius aumtus	550mL	1ton	Zhejiang	Zhejiang	2022/6/18
	Ctenopharyngodon idellus	550mL	1ton	Zhejiang	Zhejiang	2022/6/18
	Ophiocephalus argus	550mL	1ton	Zhejiang	Zhejiang	2022/6/18
	Hypophthalmichthys nobilis	550mL	1ton	Jiangxi	Zhejiang	2022/6/18
	Ophiocephalus argus	550mL	1ton	Zhejiang	Zhejiang	2022/6/18
	SciaemopsOcellatus	550mL	1ton	Fujian	Zhejiang	2022/6/19
	Siniperca chuatsi	550mL	1ton	Zhejiang	Zhejiang	2022/6/19
	Pseudobagrus fulvidraco	550mL	1ton	Zhejiang	Zhejiang	2022/6/19
	Larimichthys crocea	550mL	1ton	Fujian	Zhejiang	2022/6/19
	Ophiocephalus argus	550mL	2ton	Foshan	Fujian	2022/6/16
	Siniperca chuatsi	550mL	1ton	Fujian	Fujian	2022/6/16
	Lateolabrax japonicus	550mL	2ton	Fujian	Fujian	2022/6/16
	Siniperca chuatsi	550mL	2ton	Guangzhou	Fujian	2022/6/17
	Lateolabrax japonicus	550mL	2ton	Guangzhou	Fujian	2022/6/17
July	*Scophthalmus maximus*	550mL	1000L	Shanghai	Shanghai	2022/7/13
	Megalobrama amblycephala	550mL	1000L	Shanghai	Shanghai	2022/7/13
	Ophiocephalus argus	550mL	1000L	Shanghai	Shanghai	2022/7/13
	Mugil cephalus	550mL	1000L	Shanghai	Shanghai	2022/7/13
	Carassius aumtus	550mL	1000L	Shanghai	Shanghai	2022/7/13
	Hypophthalmichthys nobilis	550mL	1000L	Shanghai	Shanghai	2022/7/13
	Hypophthalmichthys nobilis	550mL	1000L	Shanghai	Shanghai	2022/7/13
	Ctenopharyngodon idellus	550mL	1000L	Shanghai	Shanghai	2015/7/14

	Carassius aumtus	550mL	1000L	Shanghai	Shanghai	2015/7/14
	Siniperca chuatsi	550mL	5ton	Guangdong	Zhejiang	2022/7/13
	Lateolabrax japonicus	550mL	5ton	Zhejiang	Zhejiang	2022/7/13
	Ophiocephalus argus	550mL	100L	Jiangsu	Zhejiang	2022/7/13
	Lateolabrax japonicus	550mL	5ton	Zhejiang	Zhejiang	2022/7/13
	Pseudobagrus fulvidraco	550mL	5ton	Zhejiang	Zhejiang	2022/7/13
	Pseudobagrus fulvidraco	550mL	5ton	Bozhou,Anhui	Zhejiang	2022/7/13
	Hemiculter leucisculus	550mL	5ton	Changzhou,Jiangsu	Zhejiang	2022/7/13
	Lateolabrax japonicus	550mL	5ton	Zhejiang	Zhejiang	2022/7/14
	Metapenaeus ensis	550mL	5ton	Taizhou,Zhejiang	Zhejiang	2022/7/14
	Macrobrachium rosenbergii	550mL	5ton	Ningbo,Zhejiang	Zhejiang	2022/7/14
	Ctenopharyngodon idellus	550mL	5ton	Jiangxi	Zhejiang	2022/7/14
	Carassius aumtus	550mL	5ton	Yancheng,Jiangsu	Zhejiang	2022/7/14
	Carassius aumtus	550mL	5ton	Jiangsu	Zhejiang	2022/7/14
	Ophiocephalus argus	550mL	5ton	Zhejiang	Zhejiang	2022/7/14
	Lateolabrax japonicus	550mL	5ton	Suqian,Jiangsu	Zhejiang	2022/7/14
	Hypophthalmichthys molitrix	550mL	5ton	Pingxiang,Jiangsu	Zhejiang	2022/7/14
	Carassius aumtus	550mL	5ton	Jiangsu	Zhejiang	2022/7/14
	Epinephelussp	550mL	5ton	Fujian	Fujian	2022/7/9
	Pagrosomus major	550mL	5ton	Fujian	Fujian	2022/7/9
	Epinephelussp	550mL	5ton	Fujian	Fujian	2022/7/9
	Larimichthys crocea	550mL	5ton	Fujian	Fujian	2022/7/9
	Scophthalmus maximus	550mL	5ton	Shandong	Fujian	2022/7/9
	Lateolabrax japonicus	550mL	5ton	Fujian	Fujian	2022/7/9
	Epinephelussp	550mL	5ton	Fujian	Fujian	2022/7/10
	Scophthalmus maximus	550mL	5ton	Fujian	Fujian	2022/7/10
	Scophthalmus maximus	550mL	5ton	Shandong	Fujian	2022/7/10
	Scophthalmus maximus	550mL	5ton	Shandong	Fujian	2022/7/10
	Megalobrama amblycephala	550mL	5ton	Fujian	Fujian	2022/7/10
August	*Lateolabrax japonicus*	550mL	20ton	Shanghai	Shanghai	2022/8/19
	Megalobrama amblycephala	550mL	10ton	Jiangsu	Shanghai	2022/8/19
	Carassius aumtus	550mL	20ton	Zhejiang	Shanghai	2022/8/19
	Hypophthalmichthys nobilis	550mL	20ton	Jiangsu	Shanghai	2022/8/19
	Ophiocephalus argus	550mL	20ton	Shandong	Shanghai	2022/8/19
	Pseudobagrus fulvidraco	550mL	20ton	Anhui	Shanghai	2022/8/19
	Pseudobagrus fulvidraco	550mL	20ton	Jiangsu	Shanghai	2022/8/19
	Siniperca chuatsi	550mL	10ton	Guangdong	Shanghai	2022/8/19
	Lateolabrax japonicus	550mL	20ton	Shanghai	Shanghai	2022/8/19
	Scophthalmus maximus	550mL	10ton	Fujian	Shanghai	2022/8/19
	Larimichthys crocea	550mL	20ton	Fujian	Zhejiang	2022/8/19
	Carassius aumtus	550mL	20ton	Jiangsu	Zhejiang	2022/8/19
	Larimichthys crocea	550mL	20ton	Fujian	Zhejiang	2022/8/19
	Ctenopharyngodon idellus	550mL	20ton	Zhejiang	Zhejiang	2022/8/19

	Lateolabrax japonicus	550mL	20ton	Zhejiang	Zhejiang	2022/8/20
	Scophthalmus maximus	550mL	20ton	Shandong	Zhejiang	2022/8/20
	Ctenopharyngodon idellus	550mL	20ton	Jiangxi	Zhejiang	2022/8/20
	Hypophthalmichthys nobilis	550mL	20ton	Jiangxi	Zhejiang	2022/8/20
	Hypophthalmichthys nobilis	550mL	20ton	Jiangsu	Zhejiang	2022/8/20
	Ophiocephalus argus	550mL	20ton	Zhejiang	Zhejiang	2022/8/20
	Ophiocephalus argus	550mL	20ton	Zhejiang	Zhejiang	2022/8/20
	Epinephelussp	550mL	20ton	Shandong	Zhejiang	2022/8/21
	Epinephelussp	550mL	2000L	Fujian	Fujian	2022/8/18
	Penaeus monodon	550mL	1000L	Fujian	Fujian	2022/8/18
	Carassius aumtus	550mL	2000L	Fujian	Fujian	2022/8/19
	Hypophthalmichthys nobilis	550mL	2000L	Fujian	Fujian	2022/8/19
	Ctenopharyngodon idellus	550mL	2000L	Fujian	Fujian	2022/8/19
	Epinephelussp	550mL	2000L	Fujian	Fujian	2022/8/20
	Sciaenops ocellatus	550mL	2000L	Fujian	Fujian	2022/8/20
	Pagrosomus major	550mL	2000L	Fujian	Fujian	2022/8/20
	Pampus sinensis	550mL	2000L	Fujian	Fujian	2022/8/20
	Lateolabrax japonicus	550mL	2000L	Fujian	Fujian	2022/8/20
September	*Carassius aumtus*	550mL	10ton	Shanghai	Shanghai	2022/9/23
	Pseudobagrus fulvidraco	550mL	10ton	Shanghai	Shanghai	2022/9/23
	Lateolabrax japonicus	550mL	10ton	Shanghai	Shanghai	2022/9/23
	Pseudobagrus fulvidraco	550mL	10ton	Jiangsu	Shanghai	2022/9/23
	Pseudobagrus fulvidraco	550mL	10ton	Zhejiang	Shanghai	2022/9/23
	Erythroculter ilishaeformis	550mL	10ton	Zhejiang	Shanghai	2022/9/23
	Penaeus vannamei Boone	550mL	10ton	Jiangsu	Shanghai	2022/9/23
	Penaeus monodon	550mL	10ton	Jiangsu	Shanghai	2022/9/23
	Ietalurus Punetaus	550mL	10ton	Anhui	Shanghai	2022/9/23
	Oxyeleotris marmorata	550mL	10ton	Guandgong	Shanghai	2022/9/23
	Ctenopharyngodon idellus	550mL	2000L	Jiangsu	Zhejiang	2022/9/23
	Carassius aumtus	550mL	1000L	Zhejiang	Zhejiang	2022/9/23
	Hypophthalmichthys nobilis	550mL	2000L	Zhejiang	Zhejiang	2022/9/23
	Lateolabrax japonicus	550mL	2000L	Jiangsu	Zhejiang	2022/9/23
	Pagrosomus major	550mL	2000L	Zhejiang	Zhejiang	2022/9/23
	Lateolabrax japonicus	550mL	2000L	Zhejiang	Zhejiang	2022/9/24
	Siniperca chuatsi	550mL	2000L	Zhejiang	Zhejiang	2022/9/24
	Ctenopharyngodon idellus	550mL	2000L	Jiangsu	Zhejiang	2022/9/24
	Hypophthalmichthys nobilis	550mL	2000L	Zhejiang	Zhejiang	2022/9/24
	Mylopharyngdon piceus Richardson	550mL	2000L	Jiangsu	Zhejiang	2022/9/24
	Pseudobagrus fulvidraco	550mL	10ton	Fujian	Fujian	2022/9/23
	Carassius aumtus	550mL	10ton	Fujian	Fujian	2022/9/23
	Siniperca chuatsi	550mL	10ton	Fujian	Fujian	2022/9/23
	Tinca tinca	550mL	10ton	Fujian	Fujian	2022/9/23

	Scophthalmus maximus	550mL	10ton	Fujian	Fujian	2022/9/23
	Siniperca chuatsi	550mL	10ton	Fujian	Fujian	2022/9/24
	Scophthalmus maximus	550mL	10ton	Shandong	Fujian	2022/9/24
	Trachinotus ovatus	550mL	10ton	Fujian	Fujian	2022/9/24
	Epinephelussp	550mL	10ton	Fujian	Fujian	2022/9/24
	Lateolabrax japonicus	550mL	10ton	Guangdong	Fujian	2022/9/24
October	*Macrobra'chiumrosenbergiideman*	550mL	10ton	Shanghai	Shanghai	2022/10/22
	Pseudobagrus fulvidraco	550mL	10ton	Zhejiang	Shanghai	2022/10/22
	Penaeus monodon	550mL	10ton	Shanghai	Shanghai	2022/10/22
	Pseudobagrus fulvidraco	550mL	10ton	Jiangsu	Shanghai	2022/10/22
	Lateolabrax japonicus	550mL	10ton	Guangdong	Shanghai	2022/10/22
	Ophiocephalus argus	550mL	10ton	Zhejiang	Shanghai	2022/10/22
	Carassius aumtus	550mL	10ton	Jiangsu	Shanghai	2022/10/23
	Penaeus vannamei Boone	550mL	10ton	Zhejiang	Shanghai	2022/10/23
	Cyprinus carpio	550mL	10ton	Jiangsu	Shanghai	2022/10/23
	Carassius aumtus	550mL	10ton	Jiangsu	Shanghai	2022/10/23
	Ophiocephalus argus	550mL	10ton	Shandong	Zhejiang	2022/10/26
	Lateolabrax japonicus	550mL	10ton	Zhejiang	Zhejiang	2022/10/26
	Pseudobagrus fulvidraco	550mL	10ton	Anhui	Zhejiang	2022/10/26
	Siniperca chuatsi	550mL	10ton	Guangdong	Zhejiang	2022/10/26
	Carassius aumtus	550mL	10ton	Zhejiang	Zhejiang	2022/10/26
	Siniperca chuatsi	550mL	10ton	Guangdong	Zhejiang	2022/10/26
	Lateolabrax japonicus	550mL	10ton	Zhejiang	Zhejiang	2022/10/26
	Pagrosomus major	550mL	10ton	Fujian	Zhejiang	2022/10/26
	Scophthalmus maximus	550mL	10ton	Fujian	Zhejiang	2022/10/26
	Hypoplectrus indigo	550mL	10ton	Guangdong	Zhejiang	2022/10/26
	Epinephelussp	550mL	2ton	Shanghai	Fujian	2022/10/26
	Sciaenops ocellatus	550mL	2ton	Jiangsu	Fujian	2022/10/26
	Ctenopharyngodon idellus	550mL	2ton	Zhejiang	Fujian	2022/10/27
	Hypophthalmichthys nobilis	550mL	2ton	Jiangsu	Fujian	2022/10/27
	Carassius aumtus	550mL	2ton	Shandong	Fujian	2022/10/27
	Trachinotus ovatus	550mL	2ton	Anhui	Fujian	2022/10/28
	Lateolabrax japonicus	550mL	2ton	Jiangsu	Fujian	2022/10/28
	Epinephelussp	550mL	2ton	Guangdong	Fujian	2022/10/28
	Pagrosomus major	550mL	2ton	Shanghai	Fujian	2022/10/28
	Sciaenops ocellatus	550mL	2ton	Fujian	Fujian	2022/10/28

Table S12 The 2022 Temporary Water Samples Sampling Record Form

Sampling month	Name	Sampling amount	Base	place of origin	sampling area	sampling date
April	*Erythroculter ilishaeformis*	550mL	1ton	Shanghai	Shanghai	2022/4/15
	Lateolabrax japonicus	550mL	1ton	Shanghai	Shanghai	2022/4/15

	Pseudobagrus fulvidraco	550mL	1ton	Zhejiang	Shanghai	2022/4/15
	Ophiocephalus argus	550mL	1ton	Shanghai	Shanghai	2022/4/15
	Carassius aumtus	550mL	1ton	Shanghai	Shanghai	2022/4/15
	Scophthalmus maximus	550mL	1ton	Shandong	Shanghai	2022/4/15
	Leiocassis longirostris	550mL	1ton	Jiangsu	Shanghai	2022/4/15
	Oreochromis spp	550mL	1ton	Jiangsu	Shanghai	2022/4/15
	Lateolabrax japonicus	550mL	1ton	Shanghai	Shanghai	2022/4/15
	Siniperca chuatsi	550mL	1ton	Guangzhou	Shanghai	2022/4/15
	Ophiocephalus argus	550mL	1ton	Anhui	Shanghai	2022/4/15
	Carassius aumtus	550mL	1ton	Dianshan Lake,Shanghai	Shanghai	2022/4/16
	Hypophthalmichthys molitrix	550mL	1ton	Shanghai	Shanghai	2022/4/16
	Carassius aumtus	550mL	1ton	Chongming Island,Shanghai	Shanghai	2022/4/16
	Ctenopharyngodon idellus	550mL	1ton	Sheyang,Jiangsu	Shanghai	2022/4/16
	Pampus sinensis	350mL	1ton	Fujian	Fujian	2022/4/14
	Scophthalmus maximus	350mL	1ton	Shandong	Fujian	2022/4/14
	Siniperca chuatsi	550mL	1ton	Ningbo	Zhejiang	2022/4/16
	Pagrosomus major	550mL	1ton	Ningbo	Zhejiang	2022/4/16
	Ophiocephalus argus, Carassius aumtus	550mL	1ton	Ningbo	Zhejiang	2022/4/16
May	*Pseudobagrus fulvidraco*	550mL	0.5ton	Shanghai	Shanghai	2022/5/18
	Mugil cephalus	550mL	0.5ton	Shanghai	Shanghai	2022/5/18
	Ophiocephalus argus	550mL	0.5ton	Shanghai	Shanghai	2022/5/18
	Penaeus monodon	550mL	0.5ton	Shanghai	Shanghai	2022/5/18
	Scophthalmus maximus	550mL	0.5ton	Shandong	Shanghai	2022/5/18
	Siniperca chuatsi	550mL	0.5ton	Guangzhou	Shanghai	2022/5/18
	Lateolabrax japonicus	550mL	0.5ton	Guangzhou	Shanghai	2022/5/18
	Epinephelussp	550mL	0.5ton	Fujian	Shanghai	2022/5/18
	Ietalurus Punetaus	550mL	0.5ton	Jiangsu	Shanghai	2022/5/18
	Megalobrama amblycephala	550mL	0.5ton	Shanghai	Shanghai	2022/5/18
	Ctenopharyngodon idellus	550mL	0.5ton	Shanghai	Shanghai	2022/5/19
	Hypophthalmichthys nobilis	550mL	0.5ton	Shanghai	Shanghai	2022/5/19
	Pseudobagrus fulvidraco	550mL	0.5ton	Shanghai	Shanghai	2022/5/19
	Lateolabrax japonicus	550mL	0.5ton	Guangzhou	Shanghai	2022/5/19
	Ophiocephalus argus	550mL	0.5ton	Shanghai	Shanghai	2022/5/19
	Carassius aumtus	550mL	10ton	Jiangsu	Zhejiang	2022/5/18
	Megalobrama amblycephala	550mL	1ton	Jiangxi	Zhejiang	2022/5/18
	Ctenopharyngodon idellus	550mL	0.5ton	Jiangsu	Zhejiang	2022/5/18
	Lateolabrax japonicus	550mL	10ton	Zhejiang	Zhejiang	2022/5/18
	Carassius aumtus	550mL	5ton	Jiangsu	Zhejiang	2022/5/18
	Hypophthalmichthys nobilis	550mL	10ton	Anhui	Zhejiang	2022/5/18
	Megalobrama amblycephala	550mL	2ton	Zhejiang	Zhejiang	2022/5/18

	Ophiocephalus argus	550mL	2ton	Shanghai	Zhejiang	2022/5/18
	Ophiocephalus argus	550mL	1ton	Zhejiang	Zhejiang	2022/5/18
	Carassius aumtus	550mL	0.5ton	Jiangsu	Zhejiang	2022/5/18
	Ophiocephalus argus	550mL	0.5ton	Zhejiang	Zhejiang	2022/5/19
	Pseudobagrus fulvidraco	550mL	0.5ton	Anhui	Zhejiang	2022/5/19
	Ophiocephalus argus	550mL	1ton	Anhui	Zhejiang	2022/5/19
	Oreochromis spp	550mL	0.5ton	Zhejiang	Zhejiang	2022/5/19
	Megalobrama amblycephala	550mL	1ton	Zhejiang	Zhejiang	2022/5/19
	Lateolabrax japonicus (bottle X2)	550mL	2ton	Xiamen	Fujian	2022/5/18
	Pampus sinensis （bottle X4）	550mL	2ton	Xiamen	Fujian	2022/5/18
	Pagrosomus major （bottle X6）	550mL	2ton	Xiamen	Fujian	2022/5/18
	Penaeus vannamei （X8）	550mL	2ton	Xiamen	Fujian	2022/5/18
	Larimichthys crocea 105 （bottle X10）	550mL	2ton	Xiamen	Fujian	2022/5/18
	Larimichthys crocea 106 （bottle F2）	550mL	2ton	Fuzhou	Fujian	2022/5/19
	Scophthalmus maximus （bottle F3）	550mL	2ton	Fuzhou	Fujian	2022/5/19
	Megalobrama amblycephala (bottle F3)	550mL	2ton	Fuzhou	Fujian	2022/5/19
	Lateolabrax japonicus （bottle F7）	550mL	2ton	Fuzhou	Fujian	2022/5/19
	Siniperca chuatsi （bottle F8）	550mL	2ton	Fuzhou	Fujian	2022/5/19
	Pseudobagrus fulvidraco （bottle F10）	550mL	2ton	Fuzhou	Fujian	2022/5/19
	Hypophthalmichthys nobilis （bottle F12）	550mL	2ton	Fuzhou	Fujian	2022/5/20
	Hypophthalmichthys molitrix （bottle F14）	550mL	2ton	Fuzhou	Fujian	2022/5/20
	Ctenopharyngodon idellus （bottle F16）	550mL	2ton	Fuzhou	Fujian	2022/5/20
	Carassius aumtus （bottle F18）	550mL	2ton	Fuzhou	Fujian	2022/5/20
June	*Hypophthalmichthys molitrix*	550mL	2000L	Shanghai	Shanghai	2022/6/17
	Ctenopharyngodon idellus	550mL	2000L	Shanghai	Shanghai	2022/6/17
	Hypophthalmichthys nobilis	550mL	2000L	Shanghai	Shanghai	2022/6/17
	Carassius aumtus	550mL	2000L	Shanghai	Shanghai	2022/6/17
	Ophiocephalus argus	550mL	1000L	Shanghai	Shanghai	2022/6/17

	Scophthalmus maximus	550mL	2000L	Shanghai	Shanghai	2022/6/18
	Siniperca chuatsi	550mL	2000L	Shanghai	Shanghai	2022/6/18
	Pseudobagrus fulvidraco	550mL	1000L	Shanghai	Shanghai	2022/6/18
	Mugil cephalus	550mL	1000L	Shanghai	Shanghai	2022/6/18
	Ophiocephalus argus	550mL	2000L	Shanghai	Shanghai	2022/6/18
	Pampus sinensis	550mL	2000L	Shanghai	Shanghai	2022/6/18
	Epinephelussp	550mL	1000L	Shanghai	Shanghai	2022/6/18
	Carassius aumtus	550mL	1000L	Shanghai	Shanghai	2022/6/18
	Megalobrama amblycephala	550mL	1000L	Shanghai	Shanghai	2022/6/18
	Lateolabrax japonicus	550mL	2000L	Shanghai	Shanghai	2022/6/18
	Ctenopharyngodon idellus	550mL	2ton	Jiangsu	Zhejiang	2022/6/17
	Hypophthalmichthys nobilis	550mL	2ton	Jiangsu	Zhejiang	2022/6/17
	Hypophthalmichthys nobilis	550mL	2ton	Jiangxi	Zhejiang	2022/6/17
	Scophthalmus maximus	550mL	2ton	Jiangsu	Zhejiang	2022/6/18
	Siniperca chuatsi	550mL	2ton	Zhejiang	Zhejiang	2022/6/18
	Lateolabrax japonicus	550mL	2ton	Zhejiang	Zhejiang	2022/6/18
	Carassius aumtus	550mL	2ton	Zhejiang	Zhejiang	2022/6/18
	Ctenopharyngodon idellus	550mL	2ton	Zhejiang	Zhejiang	2022/6/18
	Ophiocephalus argus	550mL	2ton	Zhejiang	Zhejiang	2022/6/18
	Hypophthalmichthys nobilis	550mL	2ton	Jiangxi	Zhejiang	2022/6/18
	Ophiocephalus argus	550mL	2ton	Zhejiang	Zhejiang	2022/6/18
	SciaemopsOcellatus	550mL	2ton	Fujian	Zhejiang	2022/6/19
	Siniperca chuatsi	550mL	2ton	Zhejiang	Zhejiang	2022/6/19
	Pseudobagrus fulvidraco	550mL	2ton	Zhejiang	Zhejiang	2022/6/19
	Larimichthys crocea	550mL	2ton	Fujian	Zhejiang	2022/6/19
	Scophthalmus maximus	550mL	2ton	Shandong	Fujian	2022/6/16
	Mugil cephalus	550mL	2ton	Fujian	Fujian	2022/6/16
	Megalobrama amblycephala	550mL	1ton	Fujian	Fujian	2022/6/16
	Carassius aumtus	550mL	2ton	Fujian	Fujian	2022/6/16
	Pseudobagrus fulvidraco	550mL	1ton	Guangzhou	Fujian	2022/6/17
	Megalobrama amblycephala	550mL	1ton	Guangzhou	Fujian	2022/6/17
	Scophthalmus maximus	550mL	2ton	Xiamen	Fujian	2022/6/17
	Cyprinus carpio	550mL	1ton	Shandong	Fujian	2022/6/17
	Leiocassis longirostris	550mL	2ton	Shandong	Fujian	2022/6/17
	Acipensersinensis	550mL	3ton	Nanning	Fujian	2022/6/17
July	*Scophthalmus maximus*	550mL	2000L	Shanghai	Shanghai	2022/7/13
	Epinephelussp	550mL	2000L	Shanghai	Shanghai	2022/7/13
	Megalobrama amblycephala	550mL	2000L	Shanghai	Shanghai	2022/7/13
	Siniperca chuatsi	550mL	2000L	Shanghai	Shanghai	2022/7/13
	Ophiocephalus argus	550mL	2000L	Shanghai	Shanghai	2022/7/13
	Mugil cephalus	550mL	2000L	Shanghai	Shanghai	2022/7/13
	Carassius aumtus	550mL	2000L	Shanghai	Shanghai	2022/7/13
	Hypophthalmichthys nobilis	550mL	2000L	Shanghai	Shanghai	2022/7/13

Pagrosomus major	550mL	2000L	Shanghai	Shanghai	2022/7/13
Pampus sinensis	550mL	2000L	Shanghai	Shanghai	2022/7/13
Hypophthalmichthys nobilis	550mL	2000L	Shanghai	Shanghai	2022/7/14
Ctenopharyngodon idellus	550mL	2000L	Shanghai	Shanghai	2022/7/14
Carassius aumtus	550mL	2000L	Shanghai	Shanghai	2022/7/14
Siniperca chuatsi	550mL	1000L	Shanghai	Shanghai	2022/7/14
Lateolabrax japonicus	550mL	1000L	Shanghai	Shanghai	2022/7/14
Lateolabrax japonicus	550mL	100L	Zhejiang	Zhejiang	2022/7/13
Siniperca chuatsi	550mL	100L	Guangdong	Zhejiang	2022/7/13
Pseudobagrus fulvidrac	550mL	100L	Jiangsu	Zhejiang	2022/7/13
Mugil cephalus	550mL	100L	Zhejiang	Zhejiang	2022/7/13
Hypophthalmichthys nobilis	550mL	100L	Zhejiang	Zhejiang	2022/7/13
Carassius aumtus	550mL	100L	Jiangsu	Zhejiang	2022/7/13
Pagrosomus major	550mL	100L	Fujian	Zhejiang	2022/7/14
Siniperca chuatsi	550mL	100L	Guangdong	Zhejiang	2022/7/14
Megalobrama amblycephala	550mL	100L	Jiangsu	Zhejiang	2022/7/14
Lateolabrax japonicus	550mL	100L	Zhejiang	Zhejiang	2022/7/14
Carassius aumtus	550mL	100L	Zhejiang	Zhejiang	2022/7/14
Epinephelussp	550mL	100L	Guangdong	Zhejiang	2022/7/14
Scophthalmus maximus	550mL	100L	Fujian	Zhejiang	2022/7/14
Lateolabrax japonicus	550mL	100L	Zhejiang	Zhejiang	2022/7/14
Epinephelussp	550mL	2ton	Fujian	Fujian	2022/7/9
Pagrosomus major	550mL	2ton	Fujian	Fujian	2022/7/9
Epinephelussp	550mL	2ton	Fujian	Fujian	2022/7/9
Larimichthys crocea	550mL	2ton	Fujian	Fujian	2022/7/9
Scophthalmus maximus	550mL	2ton	Shandong	Fujian	2022/7/9
Lateolabrax japonicus	550mL	2ton	Fujian	Fujian	2022/7/9
Epinephelussp	550mL	2ton	Fujian	Fujian	2022/7/10
Scophthalmus maximus	550mL	2ton	Fujian	Fujian	2022/7/10
Scophthalmus maximus	550mL	2ton	Shandong	Fujian	2022/7/10
Scophthalmus maximus	550mL	2ton	Shandong	Fujian	2022/7/10
Megalobrama amblycephala	550mL	2ton	Fujian	Fujian	2022/7/10
